# Framing the Salmonidae Family Phylogenetic Portrait: A More Complete Picture from Increased Taxon Sampling

**DOI:** 10.1371/journal.pone.0046662

**Published:** 2012-10-05

**Authors:** Alexis Crête-Lafrenière, Laura K. Weir, Louis Bernatchez

**Affiliations:** 1 Institut de Biologie Intégrative et des Systèmes, Pavillon Charles-Eugène-Marchand, Université Laval, Québec, Québec, Canada; 2 Department of Zoology, University of British Columbia, Vancouver, British Columbia, Canada; Consiglio Nazionale delle Ricerche (CNR), Italy

## Abstract

Considerable research efforts have focused on elucidating the systematic relationships among salmonid fishes; an understanding of these patterns of relatedness will inform conservation- and fisheries-related issues, as well as provide a framework for investigating evolutionary mechanisms in the group. However, uncertainties persist in current Salmonidae phylogenies due to biological and methodological factors, and a comprehensive phylogeny including most representatives of the family could provide insight into the causes of these difficulties. Here we increase taxon sampling by including nearly all described salmonid species (n = 63) to present a time-calibrated and more complete portrait of Salmonidae using a combination of molecular markers and analytical techniques. This strategy improved resolution by increasing the signal-to-noise ratio and helped discriminate methodological and systematic errors from sources of difficulty associated with biological processes. Our results highlight novel aspects of salmonid evolution. First, we call into question the widely-accepted evolutionary relationships among sub-families and suggest that Thymallinae, rather than Coregoninae, is the sister group to the remainder of Salmonidae. Second, we find that some groups in Salmonidae are older than previously thought and that the mitochondrial rate of molecular divergence varies markedly among genes and clades. We estimate the age of the family to be 59.1 MY (CI: 63.2-58.1 MY) old, which likely corresponds to the timing of whole genome duplication in salmonids. The average, albeit highly variable, mitochondrial rate of molecular divergence was estimated as ∼0.31%/MY (CI: 0.27–0.36%/MY). Finally, we suggest that some species require taxonomic revision, including two monotypic genera, *Stenodus* and *Salvethymus*. In addition, we resolve some relationships that have been notoriously difficult to discern and present a clearer picture of the evolution of the group. Our findings represent an important contribution to the systematics of Salmonidae, and provide a useful tool for addressing questions related to fundamental and applied evolutionary issues.

## Introduction

The evolutionary relationships among salmonid fishes have been the focus of extensive systematic and phylogenetic research for many decades [1]–[5]. Interest in the precise patterns of relatedness among species has been motivated by applied issues related to fisheries and conservation, as well as fundamental research involving the evolutionary processes that govern the diversification and maintenance of species [6]–[7]. Salmonid fishes offer a unique opportunity to explore a number of evolutionary and ecological concepts, including mechanisms of speciation [6], the evolution of complex life-histories [8], [9], the role of hybridization in evolution [7], patterns of chromosomal evolution [10] and genome duplication [11]. To address these evolutionary phenomena, a comprehensive salmonid phylogeny is required to carry out appropriate comparative analyses of biological diversity.

Despite the large body of work dedicated to inferring phylogenetic relationships among salmonid species, some important questions regarding their evolutionary history remain unanswered. These questions vary in their degree of resolution across different levels of biological organization, from the appropriate placement of the root of the Salmonidae tree to the role of introgression in species or subspecies designations. Unresolved issues in salmonid phylogenetics are often attributed to two causes: limitations imposed by biological factors (including parallel and convergent evolution due to similarity of ecological niches, rapid radiation, frequent hybridization, and local adaptation [4], [12]) and constraints imposed by methodological factors, including insufficient sampling of taxa or genes [5], [13], [14].

Salmonid fishes are believed to have undergone a rapid radiation between 25 and 100 million years ago following a tetraploidization event that characterizes the family [15], [16] Monophyly of Salmonidae is supported by morphological data, as are the groupings of the three subfamilies: Coregoninae (ciscoes, whitefish and inconnu), Thymallinae (grayling) and Salmoninae (huchen, lenok, trout, char and salmon) [1], [3], [17]. Based on morphological evidence, it has been suggested that Coregoninae is the sister group to the remainder of Salmonidae [3], [18], a finding that is corroborated by recent molecular investigation of some species of Salmonidae using nearly complete mitochondrial sequence data [14]. However, another recent phylogenetic study using a comprehensive set of nuclear genes suggests that Thymallinae may occupy that position [13], leaving uncertainties on the evolutionary relationship among subfamilies that has been widely accepted for decades. Moreover, recent molecular evidence indicated that the true sister group to Salmonidae may differ from those often used in phylogenetic studies [19], [20]. In light of recent ambiguity associated with both the sister group to Salmonidae and the evolutionary relationship of the three subfamilies, the position of the root remains an open question in the phylogeny of Salmonidae.

While monophyly of each of the three subfamilies remain generally unchallenged by morphological or molecular evidence, many relationships within subfamilies remain unclear. There are currently 11 recognized genera in Salmonidae, with the majority concentrated within Salmoninae (Salmoninae: *Brachymystax*, *Hucho*, *Oncorhynchus*, *Parahucho*, *Salmo*, *Salvelinus* and *Salvethymus*; Coregoninae: *Coregonus*, *Prosopium* and *Stenodus*; Thymallinae: *Thymallus*). Of these genera, three are monotypic (*Parahucho*, *Salvethymus* and *Stenodus*) and their exact position within their respective subfamilies is currently disputed. Historically, phylogenetic reconstruction of Salmoninae has placed *Parahucho perryi* in various locations in the group, including sister to *Salvelinus* [4], [21], sister to *Salmo* [5], [22] and sister to the remainder of Salmoninae [23], [24]. The other monotypic genera, *Salvethymus* in Salmoninae and *Stenodus* in Coregoninae, have unique morphologies and karyotypes that differentiate them from other genera in their respective subfamilies, despite some molecular evidence that suggests they do not warrant separate genus designation (*Salvethymus*: [4], [25]; *Stenodus*: [26]–[28]). The genus designation and position within their respective subfamilies remain unclear for the three monotypic genera.

Monotypic genera represent only one source of ambiguity in discerning evolutionary relationships within the subfamilies of Salmonidae. Most notably, the relationship among *Salvelinus*, *Oncorhynchus* and *Salmo* has been a source of considerable debate, with the long-held designation of *Oncorhynchus* and *Salmo* as sister species having been replaced with an *Oncorhynchus* and *Salvelinus* grouping based on molecular studies [5], [22], [29]. Within genera, the main issues with phylogenetic reconstruction are concentrated in the three more speciose genera, *Oncorhynchus*, *Salvelinus* and *Coregonus*. In *Oncorhynchus*, the position of the Japanese salmon is unclear and inconsistent across molecular studies [5], [30]–[32], and many questions arise for the relationships among the Pacific trout, which are obscured by frequent hybridization [2], [7]. Similarly, *Salvelinus* species frequently hybridize and show inconsistencies among phylogenetic studies [3], [33], [34]. In *Coregonus*, species identification presents a further obstacle in phylogenetic studies, in addition to other sources of uncertainty including parallel evolution [6], [35], phenotypic plasticity [36], recurrent trophic polymorphisms [37], [38], contemporary hybridization [7] and historical introgression [39]. Moreover, the two morphological groupings within *Coregonus*, the whitefish and ciscoes, may not constitute true monophyletic clades [27]. Thus, despite a large body of work dedicated to resolving various aspects of salmonid phylogeny, questions pertaining to many interspecific relationships persist.

Outstanding questions about relationships within Salmonidae may be resolved by increased sampling of both taxa and characters. Herein, we increase the number of species sampled to 63, doubling the number used by Stearley and Smith [3] in the most extensive morphological study performed to date and tripling the number used in the most comprehensive molecular study to date [5]. Increased sampling of taxa may subdivide long branches, allowing for a more precise resolution of phylogenetic relationships [40]–[42] and a reduction in bias associated with long-branch attraction [43], [44]. Furthermore, increased taxon sampling can be beneficial when estimating parameters for models of molecular evolution [45] and different types of phylogenetic tests including rooting analysis [46], [47], estimation of divergence times [48] and patterns of diversification [49], [50].

In addition to increasing the number of taxa sampled, we used a number of different genes to infer the salmonid phylogeny. Single gene phylogenies are inherently limited in their ability to accurately resolve relationships among taxa and are susceptible to stochastic errors. Thus, we concatenate gene sequences into a ‘supermatrix’ to strengthen the phylogenetic signal and improve node support [51]–[53]. Not all gene sequences were available for all species; however this is unlikely to have a large effect on our ability to precisely reconstruct phylogenetic relationships [54]–[56]. The simultaneous analysis of concatenated gene sequences must nonetheless be treated cautiously, as systematic errors can plague the phylogenetic inferences by strongly supporting clades that are erroneously grouped on the basis of multiple substitution artifacts (e.g., nucleotide compositional heterogeneity [57], [58], rate variation across sites [59] and rate variation across lineages [60]). Different strategies are available for detecting and minimizing non-historical signals responsible for such systematic errors, including the critical comparison of the trees resulting from parsimony and probabilistic criteria [11], functional R/Y recoding [11], [61] and increased taxon sampling [62], [63].

Our main objective was to infer the phylogeny of Salmonidae using more extensive species and character sampling. Given this new phylogeny, we attempt to address the outstanding questions regarding the evolutionary history and relationships within the group, with respect to the root of Salmonidae, the validity of monotypic genera, and patterns of relatedness among species or genera whose relationships have proved difficult to resolve. In addition, we seek to shed light on temporal aspects of salmonid evolution, including the age and divergence rates within the family.

## Results

A total of 107 DNA or tissue samples belonging to 63 salmonid species were obtained from a number of people and groups (Table S1). To suitably represent intraspecific diversity and detect non-monophyletic groups, two individuals per species were chosen from geographically distant populations or divergent lineages whenever possible. According to the groupings recommended by the Integrated Taxonomic Information System [64], our data set consisted of two *Brachymystax* species, 20 *Coregonus* species, two *Hucho* species, 11 *Oncorhynchus* species, six *Prosopium* species, six *Salmo* species, nine *Salvelinus* species, four *Thymallus* species and three species from monotypic genera: *Parahucho perryi*, *Salvethymus svetovidovi* and *Stenodus leucichthys*.

### Phylogenetic analysis of mitochondrial cytochromes

**Figure 1:**
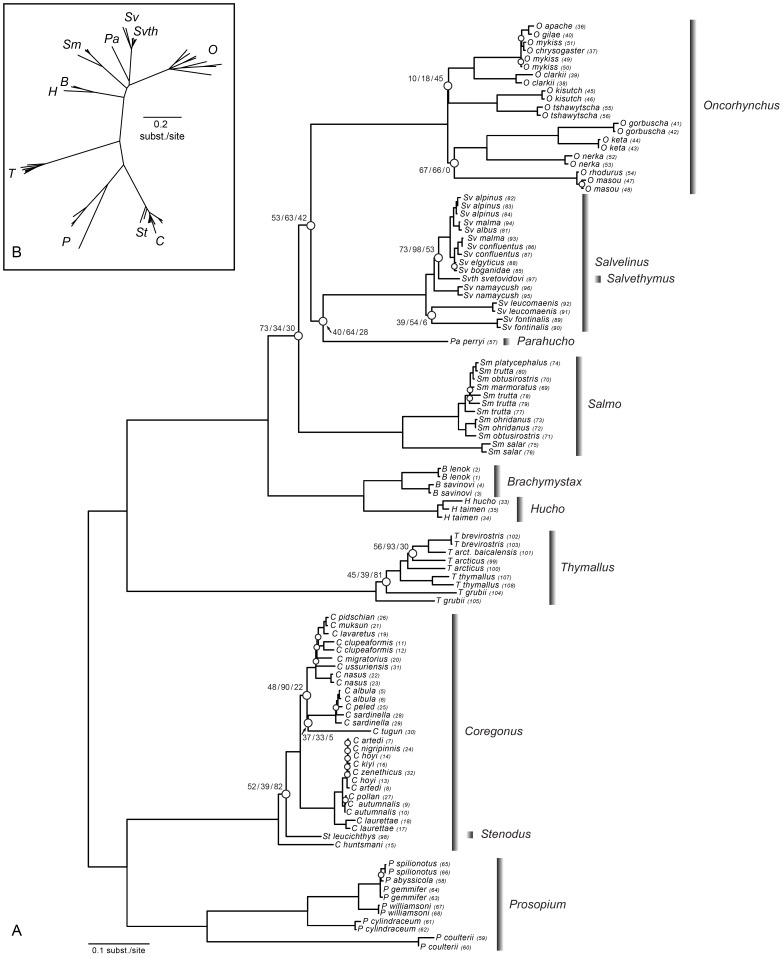
Unrooted ML phylogram based on the cytochromes data set. A: Nodes with bootstrap values less than 75% are indicated with open circles (n = 29). For some deep nodes, ML bootstrap support/BAY posterior probabilities/MP bootstrap supports are shown above the node. B: Radial view of the same tree. Abbreviations: *B* = *Brachymystax*, *C = Coregonus*, *H = Hucho*, *O = Oncorhynchus*, *Pa = Parahucho perryi*, *P = Prosopium*, *Sm = Salmo*, *Sv = Salvelinus*, *Svth = Salvethymus svetovidovi*, *St = Stenodus leucichthys* and *T = Thymallus*. Numbers beside each sample correspond to identification numbers in Table S1.

Sequences for entire cytochrome b (Cytb) and cytochrome c oxydase I (CO1) genes were obtained for all samples except *Coregonus ussuriensis*, for which only 787 bp of Cytb and 523 bp of CO1 could be amplified. Mean nucleotide frequencies were similar for CO1 and Cytb; however the third codon positions for both genes, and Cytb as a whole, showed a lack of homogeneity in nucleotide frequencies. The evolutionary model retained for each gene and for the concatenated genes data set was TIM+G+I, which justified the use of a 6 parameter model for maximum likelihood (ML) analysis and a partitioned model for Bayesian (BAY) analysis. The best trees inferred using three different approaches (maximum parsimony (MP) and two probabilistic criteria (ML, BAY)) were similar and will be interpreted in reference to the ML tree depicted in Figure 1. The nodes that were least supported in the ML analysis (bootstrap values less than 75% indicated by open circles, n = 29, Figure 1A) were the only nodes that also lacked strong support in the other two analyses (considering Bayesian posterior probabilities less than 75%). The monophyly of every genus was strongly supported in all analyses, with most of the unsupported nodes (17/29) found to be shallow in the tree and attributable to recent divergences for which the cytochrome data set might have limited resolution (open circles; Figure 1A). We show only support values for the other 12 unsupported nodes in Figure 1A as they concern deeper divergences in the tree and are the main focus of subsequent analyses.

Among the three monotypic genera only *Parahucho* did not group within another genus of the family, being weakly associated as a sister taxa to *Salvelinus* or to the clade (*Salvelinus*, *Oncorhynchus*) depending on the particular analysis. In all analyses, the monotypic genus *Salvethymus* grouped within *Salvelinus*, despite some differences in its exact position within the genus across the analytic approaches. Finally, *Stenodus* grouped within *Coregonus* in all analyses, although a position as a sister species to the remainder of the genus was also weakly supported by ML and BAY analysis. The majority of the other uncertain nodes were distributed across the different genera of the family. All cytochromes analyses indicated that the Pacific salmon formed a paraphyletic group, due to a weak association between the *Oncorhynchus kisutch* and *Oncorhynchus tshawytscha* clade with Pacific trout (*Oncorhynchus clarkii* and *Oncorhynchus mykiss*). The three other Pacific salmon species formed a weakly supported monophyletic clade with Japanese species *Oncorhynchus masou* and *Oncorhynchus rhodurus* in ML and BAY analyses; however MP analysis placed the Japanese salmon as a sister clade to the remainder of *Oncorhynchus*. MP analysis showed strong support for the position of *Salvelinus fontinalis* as a sister species to the remainder of the *Salvelinus* genus (results not shown), while the other two analyses showed weak support for a clade composed of *Salvelinus fontinalis* and *Salvelinus leucomaenis*. In *Thymallus*, *Thymallus grubii* and *Thymallus arcticus* were found to be paraphyletic, which was not the case for the latter species in the MP analysis. Two uncertain nodes were found in *Coregonus* and concerned the position of *Coregonus tugun* and the grouping of whitefish with some of the cisco species. For *Brachymystax*, *Hucho*, *Prosopium* and *Salmo*, the absence of deep unsupported nodes suggests that their evolutionary relationship was robustly inferred by the cytochromes data set.

Two unsupported nodes were found deeper in the tree and concerned the evolutionary relationship of the major genera in Salmoninae. Uncertainty arose among methods, and the following three intergeneric relationships were obtained in our cytochromes analyses:

MP: (Salmo, ((Brachymystax, Hucho), (Parahucho, (Salvelinus, Oncorhynchus))))

ML: ((Brachymystax, Hucho), (Salmo, ((Parahucho, Salvelinus), Oncorhynchus)))

BAY: (Salmo, ((Brachymystax, Hucho), ((Parahucho, Salvelinus), Oncorhynchus)))

Examination of the radial view of the tree (Figure 1B) indicates that such inconsistencies were expected; the different genera within Salmoninae are separated by short internodes deep in the tree, which constitutes a topology particularly resistant to phylogenetic inference and suggests that divergence between the genera of Salmoninae occurred during a rapid radiation event [43], [44], [65].

### Phylogenetic analysis of the gene supermatrix

We carried out phylogenetic analysis on a supermatrix, MitoNuc-NT, comprised of both mitochondrial and nuclear genes. The characteristics of the genes in MitoNuc-NT for 29 426 sites (22.9% completeness) in 33 partitions are shown in Table S2. In total, 16 mitochondrial genes (including one concatenated sequence composed of tRNA) and 17 nuclear genes were used for analysis. We detected strong compositional bias in the mitochondrial genes, which may be explained by the relatively high substitution rates inferred for these genes (Table S2). Average nucleotide frequency was similar for mitochondrial and nuclear genes, however eight mitochondrial and two nuclear genes showed compositional bias, a result that justified the RY-coding strategy in the supermatrix data sets [11], [63]. Evolutionary models and rates of molecular evolution revealed distinctive modes of evolution between nuclear and mitochondrial genes, where nuclear genes showed more symmetrical transition matrices, less variation between sites and overall slower rates, suggesting that they may be more reliable for resolving deep phylogenetic relationships.

**Figure 2:**
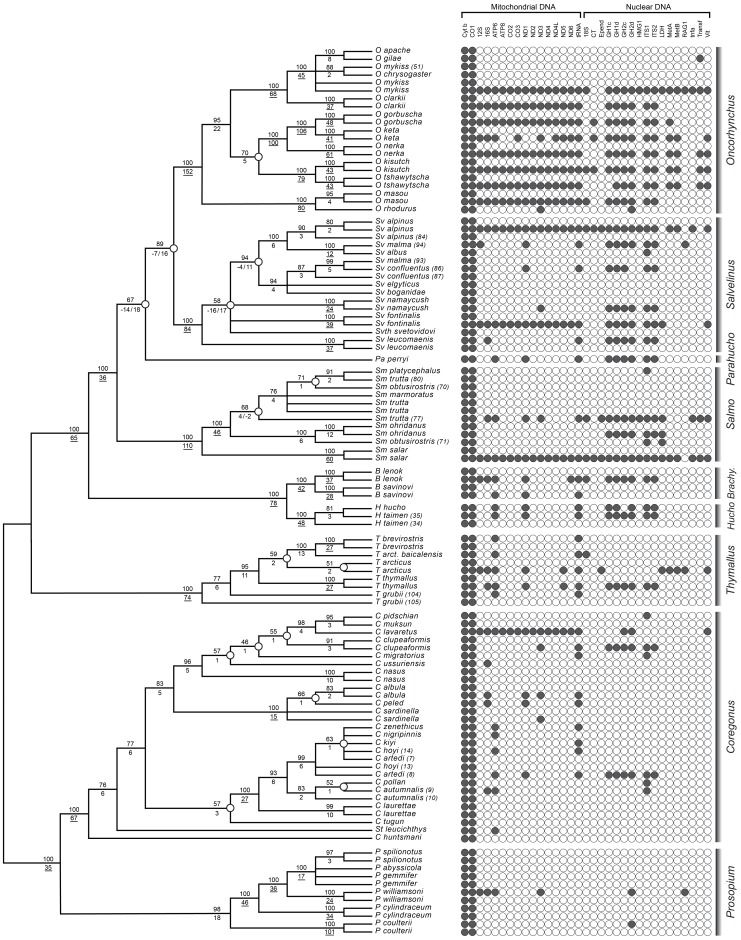
Strict consensus of 48 MP trees inferred using MitoNuc-NT showing the distribution of sequences across taxa. Bootstrap support values are indicated above branches; Bremer support indices are shown below branches. Underlined Bremer support indices indicate nodes that support significant clades. Nodes with bootstrap values less than 75% are indicated with open circles, as are nodes where conflicts between mitochondrial and nuclear genes were detected (n = 5; Bremer supports partitioned by genomic compartment are annotated in the following order: Mitochondrial/Nuclear).

**Figure 3:**
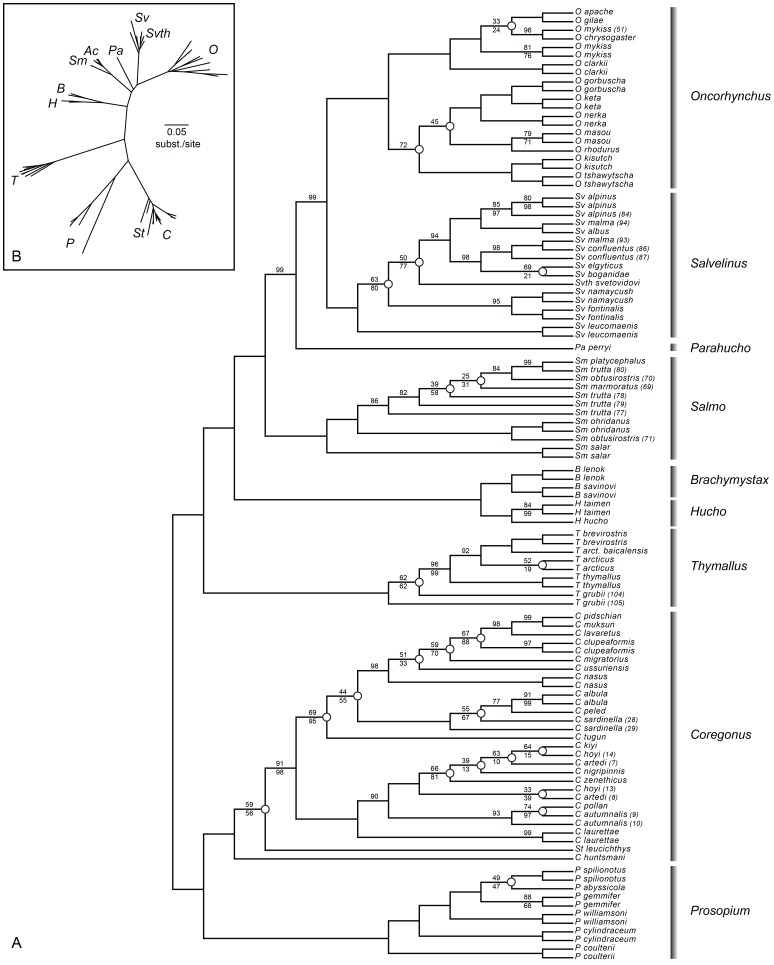
ML tree inferred by the MitoNuc-NT data set with 1 model of molecular evolution. A: Nodes with less than 75% bootstrap support are indicated by open circles (n = 24). Bootstrap values less than 100% are denoted above branches and posterior probabilities less than 100% for BAY analyses are shown under branches. B: Radial view of the same tree.

An increase in gene sampling in MitoNuc-NT relative to cytochromes resulted in improved node support for a number of relationships (Figures 2 and 3) and inferred a new evolutionary relationship for the genera in Salmoninae:

MitoNuc-NT (ALL): ((Brachymystax, Hucho), (Salmo, (Parahucho, (Salvelinus, Oncorhynchus)))).

Support for *Parahucho* as a sister taxon to (*Salvelinus*, *Oncorhynchus*) was improved in MP analysis for MitoNuc-NT (67%) over cytochromes (45%; results not shown) and was also strongly corroborated with very high bootstrap support for ML (99%) and Bayesian posterior probabilities (100%), although this node showed conflict between the mitochondrial and nuclear genes with a negative Bremer support index for the mitochondrial genome (Figure 2). Similarly, the grouping of *Salvelinus* and *Oncorhynchus* as sister taxa was much more strongly supported for MP analysis on MitoNuc-NT (89%) than the MP analysis for cytochromes (29%) and had extremely high bootstrap support in ML and BAY analyses on MitoNuc-NT (99%; Figure 3). Again, there was a conflict between genomic compartments, with a Bremer support index of −7 for the mitochondrial genes and 16 for the nuclear genes (Figure 2).

**Figure 4:**
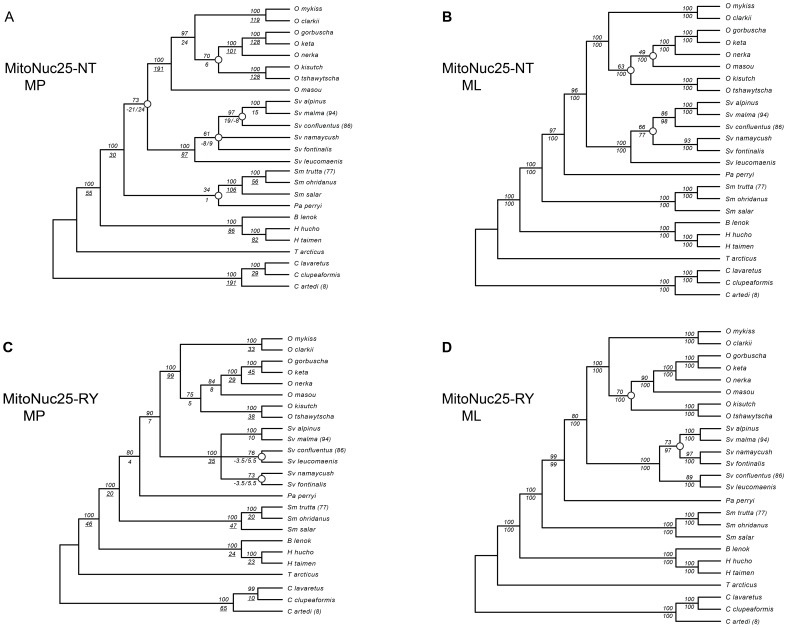
Phylogenetic analyses for MitoNuc25-NT and MitoNuc25-RY. For MP trees (A and C), bootstrap values are indicated above branches and Bremer support values are below branches. Underlined Bremer support indices indicate significant clades. Bremer support indices are partitioned by genomic compartment (Mitochondrial/Nuclear) at nodes where conflicts occur. For ML trees (B and D), bootstrap values are indicated above branches and BAY posterior probabilities are shown below branches.

To compare our results with those of Crespi and Fulton [5], we created a matrix, MitoNuc25-NT, which included only the species used in their study. Although it contained fewer taxa, this reduced data set had an increased completeness (52.4%) over MitoNuc-NT. Analyses for MitoNuc25-NT (Figure 4a and b) reflected the same sub-tree topologies as MitoNuc-NT (Figures 2 and 3), with the exception of the MP analysis that grouped *Parahucho perryi* with *Salmo*, a relationship suggested in Crespi and Fulton's [5] study. Conflicts between mitochondrial and nuclear genomes that were observed for MitoNuc-NT remained for MitoNuc25-NT, except for the node where *Parahucho perryi* was present as a sister species to (*Oncorhynchus, Salvelinus*) that was not inferred in the MitoNuc25-NT analysis (Figure 4A). Analyses on supermatrices recoded for purine/pyrimidine classification, MitoNuc-RY and MitoNuc25-RY, indicated that the purine/pyrimidine recoding allowed for some conflicts between genomic compartments to be explained by systematic errors in phylogenetic inference, rather than true contradictions between historical signals in mitochondrial and nuclear genes. Thus, tree topologies for the different approaches on MitoNuc25-RY revealed identical relationships among species with the exception of some ambiguity in MP analysis for two groups in *Salvelinus* (Figure 4C and D). Surprisingly, the same relationships within *Oncorhynchus* were resolved by both criteria, such that the outcome of MP analysis provided the same pattern as that obtained by ML analyses on both MitoNuc25-NT and MitoNuc25-RY (Figure 4B, C and D). Purine/pyrimidine recoding also allowed for the resolution of the presumed genomic conflict at the (*Salvelinus*, *Oncorhynchus*) and the (*Parahucho*, (*Salvelinus, Oncorhynchus*)) nodes. Assuming that the intergeneric relationship for Salmoninae on MitoNuc25-RY is correct (see also topology tests below), the similarities between these analyses and MP analysis for MitoNuc-NT (Figure 2) indicate that increasing the number of both taxa and genes sampled improved resolution of the phylogeny within the subfamily. By contrast, convergence of the MP and ML criteria was not observed for *Oncorhynchus* with MitoNuc-NT (Figure 2 and 3), which may indicate that more extensive sampling of genes and taxa was not sufficient to overcome the effects of stochastic and/or systematic errors in this group.

### Topology tests

Topology tests (AU and SH) were performed for two clades in which relationships varied across the different analyses: genera across Salmoninae and species within *Oncorhynchus*. AU tests may be more reliable when altering the number of taxa sampled [66], and thus we present only the results of these tests here, but show results of both AU and SH tests in Table 1. For Salmoninae, topology tests indicated that increased sampling allowed for discrimination of a single inter-generic relationship among all possible configurations. For AU tests on MitoNuc-NT, the relationship ((*Brachymystax*, *Hucho*), *Salmo*, (*Parahucho*, (*Salvelinus, Oncorhynchus*)))), obtained for all analyses on MitoNuc-NT, was the only topology that was well-supported (Table 1). Reducing the number of species in MitoNuc25-NT resulted in less discriminatory power among alternate topologies, such that AU tests suggested an alternate evolutionary relationship that mirrored that obtained for ML analyses of cytochromes (Figure 1).

**Table 1:** Results of AU and SH topology tests for subfamily Salmoninae and genus *Oncorhynchus* for different data sets; alternative topologies with significant likelihood values are shown in bold.

	Salmoninae	MitoNuc-NT		MitoNuc-RY		MitoNuc25-NT		MitoNuc25-RY	
	CONSTRAINT	AU	SH	AU	SH	AU	SH	AU	SH
1	(Salmonidae,(Salmo,(Parahucho,(Salvelinus,Oncorhynchus))))	**0.984**	**0.999**	**0.920**	**0.987**	**0.977**	**1.000**	**0.930**	**0.990**
2	(Salmonidae,(Salmo,(Oncorhynchus,(Parahucho,Salvelinus))))	0.042	**0.184**	**0.214**	**0.304**	**0.069**	**0.246**	**0.196**	**0.290**
3	(Salmonidae,(Salmo,(Salvelinus,(Parahucho,Oncorhynchus))))	0.000	0.031	0.001	**0.059**	0.000	0.043	0.000	**0.063**
4	(Salmonidae,(Parahucho,(Salvelinus,(Salmo,Oncorhynchus))))	0.000	0.000	0.001	0.007	0.000	0.000	0.002	0.009
5	(Salmonidae,(Parahucho,(Oncorhynchus,(Salmo,Salvelinus))))	0.000	0.000	0.000	0.003	0.000	0.000	0.000	0.002
6	(Salmonidae,(Parahucho,(Salmo,(Salvelinus,Oncorhynchus))))	0.011	**0.358**	**0.129**	**0.555**	0.013	**0.340**	**0.118**	**0.544**
7	(Salmonidae,(Salvelinus,(Parahucho,(Salmo,Oncorhynchus))))	0.000	0.000	0.001	0.007	0.000	0.000	0.000	0.008
8	(Salmonidae,(Salvelinus,(Oncorhynchus,(Parahucho,Salmo))))	0.000	0.000	0.000	0.003	0.000	0.000	0.000	0.002
9	(Salmonidae,(Salvelinus,(Salmo,(Parahucho,Oncorhynchus))))	0.000	0.000	0.000	0.003	0.000	0.000	0.001	0.002
10	(Salmonidae,(Oncorhynchus,(Salmo,(Parahucho,Salvelinus))))	0.000	0.001	0.013	**0.059**	0.000	0.002	0.007	0.045
11	(Salmonidae,(Oncorhynchus,(Salvelinus,(Parahucho,Salmo))))	0.000	0.000	0.000	0.003	0.000	0.000	0.000	0.002
12	(Salmonidae,(Oncorhynchus,(Parahucho,(Salmo,Salvelinus))))	0.000	0.000	0.000	0.003	0.000	0.000	0.001	0.002
13	(Salmonidae,((Oncorhynchus,Parahucho),(Salmo,Salvelinus)))	0.000	0.000	0.000	0.003	0.000	0.000	0.001	0.002
14	(Salmonidae,((Oncorhynchus,Salvelinus),(Parahucho,Salmo)))	0.040	**0.392**	**0.129**	**0.555**	0.029	**0.361**	**0.121**	**0.544**
15	(Salmonidae,((Parahucho,Salvelinus),(Salmo,Oncorhynchus)))	0.001	0.002	0.050	**0.072**	0.003	0.006	**0.052**	**0.068**

	Oncorhynchus	MitoNuc-NT		MitoNuc-RY		MitoNuc25-NT		MitoNuc25-RY	
	CONSTRAINT	AU	SH	AU	SH	AU	SH	AU	SH
1	(Salmonidae,(Trout,(Ki/T,(Masou,N/Ke/G))));	**0.937**	**0.993**	**0.941**	**0.972**	**0.899**	**0.991**	**0.941**	**0.966**
2	(Salmonidae,(Trout,(Masou,(Ki/T,N/Ke/G))));	**0.244**	**0.537**	**0.075**	**0.120**	**0.284**	**0.595**	**0.081**	**0.107**
3	(Salmonidae,(Trout,(N/Ke/G,(Ki/T,Masou))));	**0.197**	**0.509**	**0.168**	**0.177**	**0.231**	**0.555**	**0.118**	**0.127**
4	(Salmonidae,(N/Ke/G,( Trout,(Ki/T,Masou))));	**0.074**	**0.083**	**0.153**	**0.110**	**0.095**	**0.135**	**0.125**	**0.085**
5	(Salmonidae,(N/Ke/G,(Ki/T,(Trout,Masou))));	0.009	0.024	0.042	**0.050**	0.021	0.046	0.040	0.046
6	(Salmonidae,(N/Ke/G,(Masou,(Trout,Ki/T))));	0.022	0.027	0.046	**0.054**	0.026	**0.052**	**0.051**	**0.050**
7	(Salmonidae,(Masou,(N/Ke/G,(Trout,Ki/T))));	0.015	**0.050**	0.009	0.029	0.034	**0.130**	0.018	0.033
8	(Salmonidae,(Masou,( Trout,(N/Ke/G,Ki/T))));	**0.202**	**0.262**	**0.055**	0.048	**0.316**	**0.431**	**0.058**	0.049
9	(Salmonidae,(Masou,(Ki/T,(N/Ke/G, Trout))));	**0.125**	**0.119**	**0.051**	**0.054**	**0.257**	**0.298**	**0.073**	**0.062**
10	(Salmonidae,(Ki/T,(Masou,(N/Ke/G, Trout))));	0.036	0.044	0.033	**0.052**	**0.069**	**0.119**	**0.051**	**0.058**
11	(Salmonidae,(Ki/T,(N/Ke/G,(Masou, Trout)));	0.004	0.009	0.002	0.025	0.002	0.023	0.007	0.027
12	(Salmonidae,(Ki/T,( Trout,(Masou,N/Ke/G))));	0.040	**0.220**	**0.204**	**0.589**	**0.082**	**0.325**	**0.190**	**0.611**
13	(Salmonidae,((Trout,Masou),(Ki/T,N/Ke/G)));	0.005	0.043	0.034	0.044	0.006	**0.081**	0.037	0.043
14	(Salmonidae,((Trout,N/Ke/G),(Masou,Ki/T)));	**0.054**	**0.059**	**0.158**	**0.111**	**0.082**	**0.135**	**0.167**	**0.098**
15	(Salmonidae,((Trout,Ki/T),(Masou,N/Ke/G)));	0.014	**0.159**	**0.198**	**0.589**	0.026	**0.238**	**0.254**	**0.612**

Abbreviations in *Oncorhynchus*: Trout = (*O. apache*, *O. chrysogaster*, *O. clarkii*, *O. gilae*, *O. mykiss*); Ki/T = (*O. kisutch*, *O. tshawytscha*); Masou = (*O. masou*, *O. rhodurus*); N/Ke/G = (*O. nerka*, *O. keta*, *O. gorbuscha*).

Topology tests for *Oncorhynchus* suggest that the result obtained for ML analyses on MitoNuc-NT is not unanimously supported by the data, even if it was the most likely relationship obtained for this genus. Seven or more plausible alternative phylogenetic relationships were obtained for *Oncorhynchus* within each supermatrix analyzed, although the number of alternative topologies was slightly lower for the full MitoNuc-NT and -RY data sets than for the reduced MitoNuc25-NT and -RY data sets. Of the seven topologies obtained for MitoNuc-NT, three support monophyly of Pacific salmon including *Oncorhynchus masou*, while the other four suggest alternate arrangements including that obtained by MP analysis on MitoNuc-NT, as well as a topology that suggests that (*O. masou*, *Oncorhynchus rhodurus*) is a sister clade to the remainder of the genus. Despite extensive taxon and gene sampling of *Oncorhynchus*, a robust phylogenetic reconstruction of this group still remains unresolved.

### Phylogenetic relationships within genera

#### Thymallus

Analyses of both cytochromes and MitoNuc-NT revealed consistent structure within *Thymallus* species despite some weak support for certain nodes (Figures 1, 2, 3). In general, a clade consisting of *Thymallus brevirostris*, *Thymallus arcticus* and *Thymallus arcticus baicalensis* was well supported in all analyses except for MP analysis on the MitoNuc-NT data set, where bootstrap support was only 59% (Figure 2). *Thymallus thymallus* was consistently placed as a sister group to *T. brevirostris, T. arcticus, T. arcticus baicalensis*, with *Thymallus grubii* as the sister species to the remainder of the genus. These results are consistent with the most complete phylogeny of *Thymallus* species to date [67], in which both *T. grubii* and *T. arcticus* have complex and paraphyletic relationships, with the addition here of inferring a polarized evolutionary relationship for the different species in the genus.

#### Coregonus

In all analyses, the cisco species were paraphyletic, with the sardine cisco clade (*Coregonus sardinella*, *Coregonus albula* and *Coregonus peled*) grouping with the ‘true’ whitefish species. The position of *Coregonus tugun* was inconsistent and associated with weakly supported clades in all analyses. This species occurred in three different locations in the phylogeny in our analyses: 1) with the sardine cisco clade in ML and BAY analyses for cytochromes (Figure 1); 2) as a sister group to the clade comprised of the sardine cisco clade and the whitefish clade in ML and BAY analyses for MitoNuc-NT (Figure 3); 3) as a sister group to the ‘pure’ cisco group in MP analyses for all data sets (Figure 2; results not shown for cytochromes). Consequently, *C. tugun* is responsible for much of the instability within *Coregonus*, and removing this long branch from the analysis resulted in more robust relationships across the entire genus. Without *C. tugun* in the cytochrome data set, a clade uniting the sardine cisco group with whitefish species was supported with a 94% MP bootstrap value, eliminating two of the deep unsupported nodes in *Coregonus* and strongly supporting the paraphyly of ciscoes (results not shown). Following exclusion of *C. tugun*, a third problematic deep node also had increased in MP bootstrap support (92%), confirming the position of *Stenodus* nested in *Coregonus* and the position of *C. huntsmani* as a sister species to the remainder of the genus. Our analyses also support a *Coregonus artedi* complex previously identified by Turgeon and Bernatchez [39], comprised of *Coregonus artedi*, *Coregonus hoyi*, *Coregonus kiyi*, *Coregonus nigripinnis* and *Coregonus zenethicus*. Node support across whitefish species was variable, but our analyses nonetheless support the monophyly of the whitefishes.

#### Prosopium

*Prosopium* as a sister clade to *Coregonus* was unanimously supported in all analyses. Within *Prosopium* species, phylogenetic relationships were very consistent across methods and data sets, and were in accordance with previous phylogenies inferred by Bernatchez et al. [27] and Vuorinen et al. [68] (Figures 1, 2, 3). Across all analyses, uncertainty only arose for the more recently diverged Bear Lake species.

#### Brachymystax and Hucho

Species relationships within the monophyletic clades *Brachymystax* and *Hucho* were well supported in all analyses, although a strong paraphyly of *Hucho taimen* samples was found for all analyses except ML and BAY approaches for MitoNuc-NT, where monophyly of *H. taimen* was inferred, placing *Hucho hucho* as the sister species to the remainder of the genus (Figure 3).

#### Salmo

All analyses strongly supported *Salmo salar* as the sister species to the remainder of the genus. A lack of congruence among *Salmo trutta* samples and different phylogenetic positioning of the two *Salmo obtusirostris* samples resulted in poor resolution of the relationship among the remainder of the *Salmo* species. However, *Salmo ohridanus* and *Salmo obtusirostris* (sample no. 71, Table S1) formed a sister group to *Salmo trutta*, *Salmo marmoratus*, *Salmo platycephalus* and *Salmo obtusirostris* (sample no. 70, Table S1) in all analyses, despite uncertainty associated within the latter clade, which is possibly responsible for the conflict between mitochondrial and nuclear genes detected with the negative Bremer support index for the nuclear genes (Figure 2).

#### Salvelinus

MP analysis for cytochromes showed strong support for the position of *Salvelinus fontinalis* as the sister species to the remainder of the genus (results not shown), while the other two cytochromes analyses showed weak support for a clade composed of *Salvelinus fontinalis* and *Salvelinus leucomaenis* (Figure 1). By contrast, MP analysis for MitoNuc-NT was inconclusive regarding the most ancestral nodes in the genus due to an apparent conflict between genomic compartments (Figure 2) and the unresolved position of *Salvethymus svetovidovi*. ML and BAY analyses on MitoNuc-NT (Figure 3) indicated that *Salvelinus fontinalis* grouped with *Salvelinus namaycush*, although this association only obtained moderate support. Some level of conflict between genomic compartments was also detected in the most derived species of *Salvelinus* (Figure 2). In all analyses, one of the *Salvelinus malma* samples was consistently associated with *Salvelinus confluentus*, which may reflect the fact that these two species hybridize and may experience introgression [69]. Arctic charr, *Salvelinus alpinus*, grouped mainly with *Salvelinus albus* and the other *Salvelinus malma* sample, both of which originated from the Kamachatka River in Russia. A geographic complex was not comprehensively supported for *Salvelinus elgyticus* and *Salvelinus boganidae*, although these species were associated with the *Salvelinus confluentus* samples in all of our analyses.

#### Oncorhynchus

Despite the aforementioned difficulties surrounding this genus, some interspecific relationships were robustly inferred by all analyses and four main clades were consistently found: 1) the Pacific trout (*Oncorhynchus clarkii*, (*Oncorhynchus mykiss*, *Oncorhynchus apache*, *Oncorhynchus chrysogaster*, *Oncorhynchus gilae*)); 2) coho salmon and Chinook salmon (*Oncorhynchus kisutch, Oncorhynchus tshawytscha*); 3) Japanese salmon (*Oncorhynchus masou*, *Oncorhynchus rhodurus*); and 4) sockeye salmon, chum salmon and pink salmon (*Oncorhynchus nerka*, (*Oncorhynchus keta , Oncorhynchus gorbuscha*)). However, the relationships among these clades within *Oncorhynchus* were difficult to discern. All cytochromes analyses indicated that the Pacific salmon formed a paraphyletic group, due to a weak association between the *O. kisutch* and *O. tshawytscha* clade with Pacific trout (*O. clarkii* and *O. mykiss*). The three other Pacific salmon species formed a weakly supported monophyletic clade with Japanese species *O. masou* and *O. rhodurus* in ML and BAY analyses, however MP analysis of the cytochromes data set placed the Japanese salmon as a sister group to the remainder of the *Oncorhynchus*, a result supported by MP analysis on MitoNuc-NT. By contrast, ML and BAY analyses on MitoNuc-NT support monophyly of the Pacific salmon including *O. masou* and *O. rhodurus*, where the Japanese salmon are found nested within the group.

### Rooting Salmonidae

**Figure 5:**
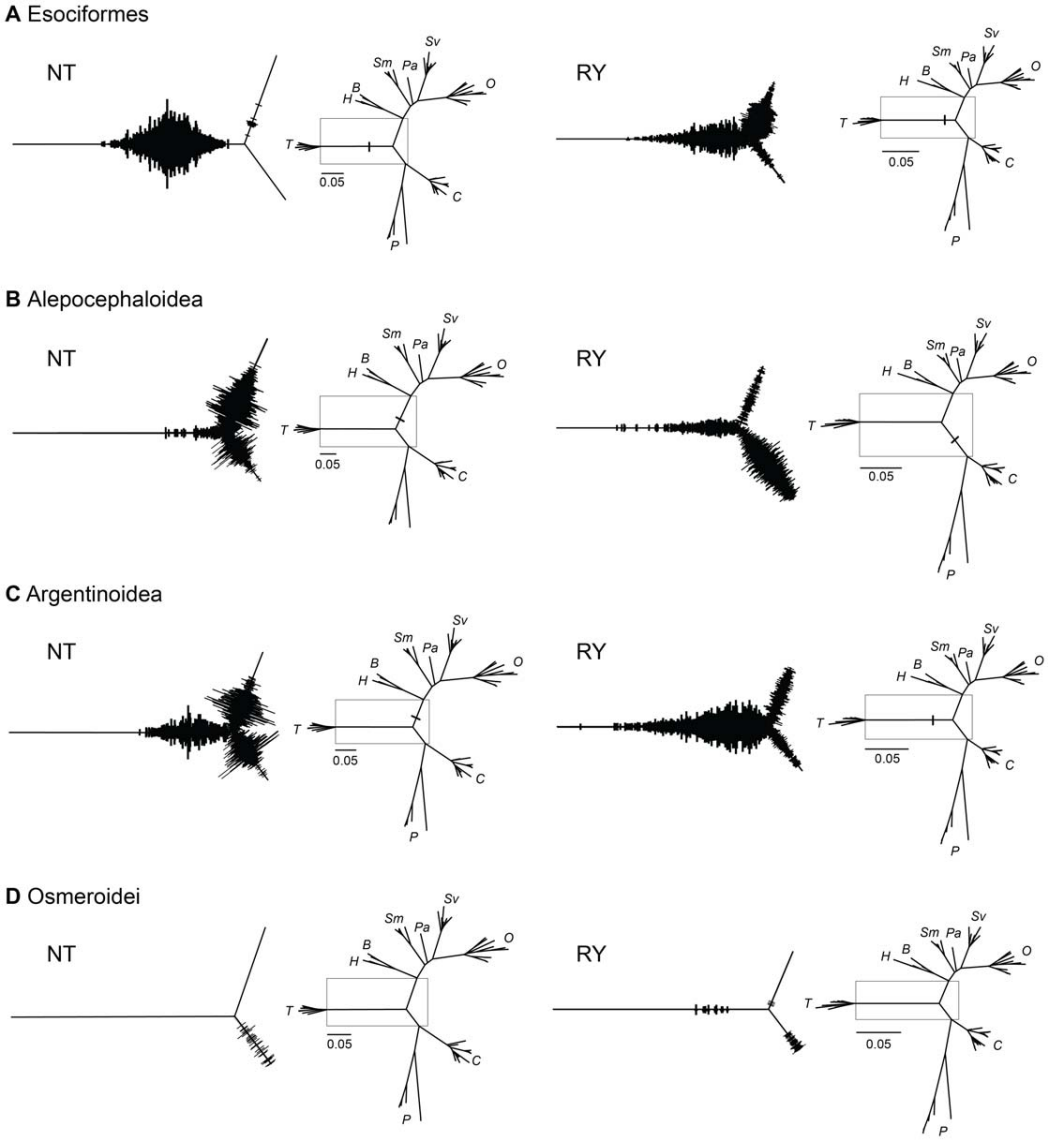
Alternative rooting for Salmonidae based on posterior probabilities of 10,000 MC^3^ trees. Boxes on radial phylograms indicated the location of the magnified areas to the left of each tree. The width of the branches indicates posterior probabilities for the position of the outgroup and the length of the branches represents the average of the posterior distributions. Trees in the left column show inferences for NT matrices; trees in the right column show inferences for RY matrices. A: Esociformes: NT 99.1% RY 57.9% (Thymallinae); NT 0.9% RY 35.2% (Salmoninae); RY 6.9% (Coregoninae); B: Alepocephaloidea: NT 3.5% RY 11.8% (Thymallinae); NT 61.6% RY 4.8% (Salmoninae); NT 34.8% RY 42.6% (Coregoninae); C: Argentinoidea: NT 22.5% RY 67.8% (Thymallinae); NT 41.4% RY 13.9% (Salmoninae); NT 33.1% RY 7.3% (Coregoninae); D: Osmeroidei: RY 0.3% (Thymallinae); RY 0.1% (Salmoninae); NET 2.2% RY 2.2% (Coregoninae).

Mitochondrial gene alignment with outgroups created less stable matrices, with 27 657 sites retained. Only four evolutionary models differed from our previous phylogenies with the addition of outgroups and concerned the following genes: CO2, ND4L, ND5 and ND6. In all analyses, inclusion of an outgroup had no effect on the structure of Salmoninae, reinforcing the evolutionary relationship inferred for MitoNuc-NT (Figure 3) and supported by the AU test (Table 1). For three of the four chosen outgroups, the majority of branch points were found at the base of one of the three subfamilies (Figure 5). However, branch points for Osmeroidei were found in multiple positions within Coregoninae, suggesting that *Galaxias m*. is not a reasonable outgroup for Salmonidae. Using Alepocephaloidea as the outgroup, the root of Salmonidae occurred either at the base of Salmoninae (MitoNuc-NT) or Coregoninae (MitoNuc-RY). Similarly, using Argentinoidea infers two different rooting structures, at the base of Salmoninae (MitoNuc-NT) or Thymallinae (MitoNuc-RY). Only Esociformes showed consistent results for NT and RY data sets, suggesting that Esociformes is a good candidate for the outgroup of Salmonidae, a result also supported by recent molecular studies [19], [20], [70]. Interestingly, using Esociformes as an outgroup inferred that the root of Salmonidae occurs at the base of Thymallinae, which is consistent with the family origin proposed by Koop et al. [13]. Despite the common inference that the root of Salmonidae occurs at the base of Coregoninae, such a configuration was not strongly supported by our analyses.

### Temporal calibration of Salmonidae

**Figure 6:**
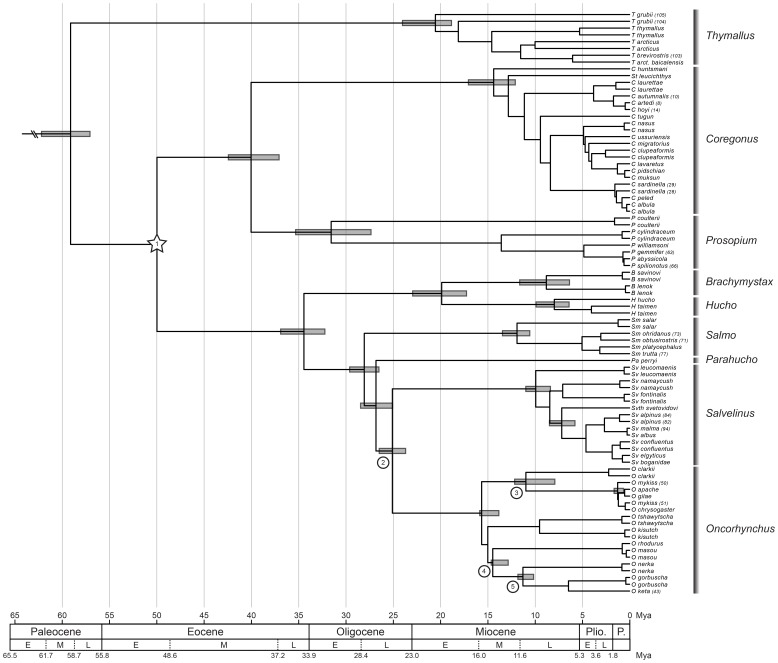
Chronogram of Salmonidae inferred on the MitoNuc-NT ML tree with a constrained fixed age of 50MY for †*Eosalmo driftwoodensis* (node 1, identified by a star). Other fossil calibration points employed as a constrained minimum age are identified by numbers in circles to the left of the appropriate nodes: †*Paleolox larsoni* (node 2); †*Oncorhynchus lacustris* (node 3); †*O. rastrotus* (node 4); †*O. keptosis* (node 5). Confidence intervals for principal divergence dates (family, subfamilies, genera and calibration points) are indicated by rectangles superimposed on the nodes indicating these divergences.

A total of 21 samples had to be excluded from the MitoNuc-NT ML tree to eliminate terminal branches of null length. A λ value of 1 was selected in the last stage of cross-validation, suggesting strong heterogeneity in evolutionary rates for the different lineages. Use of a fixed age (50 MY) for †*Eosalmo driftwoodensis* resulted in slightly older divergence times than presumed by paleontological evidence for the two other well corroborated calibration points (Figure 6). The two other fossils for which the appearance in the fossil record was not well corroborated appeared much older than their minimum estimated age: †*Oncorhynchus lacustris* (presumed age: 3.2 MY) was inferred in the Miocene (CI: 7.8–12.0 MY) and †*Paleolox larsoni* (presumed age: 11 MY) was inferred in the Oligocene (CI: 23.6–26.4 MY).

The uncertainty of the position of †*Oncorhynchus lacustris* could be due to disparity in the fossil record. Two similar fossil taxa of †*Oncorhynchus* (*Rhabdofario*) have been discovered in different geological strata: †*O. lacustris*, in the Pliocene, and †*Oncorhynchus carinatum*, at the end of the Miocene [71], [72]. These two specimens may be representatives of a same lineage, as they have similar characteristics and appear to have a similar distribution [72], [73]. Their resemblance may explain the ambiguous position of †*O. lacustris* in analyses combining morphological characters with MitoNuc-NT, which suggests three different positions for this taxon. The estimated age of 3.2 MY for †*O. lacustris* does not associate with a crown group age for the rainbow trout clade (Pleistocene CI: 1.7-0.7 MY), but rather as an older stem lineage for this group.

Despite morphological similarities with *Salvelinus* [3], [71], †*Paleolox larsoni* was assigned as a stem lineage of *Salvelinus* or *Parahucho* in analyses combining morphological characters with MitoNuc-NT. The fact that its presumed age appears too recent to reflect these divergences justifies the use of minimal temporal constraints for calibration. If †*Paleolox larsoni* truly represents a stem lineage for *Salvelinus* or *Parahucho*, and its estimated age is accurate, it is equally possible that it represents a sister species to either genus that existed millions of years after the divergence of *Salvelinus* and *Parahucho*. The nodes of the other two fossils were much closer to their estimated age: †*Oncorhynchus keptosis* (presumed age 8 MY) was inferred in the Miocene (CI: 10.2–11.8 MY), as was †*Oncorhynchus rastrosus* (presumed age 11MY; CI: 13.0–14.8 MY). This result supports the position of †*Oncorhynchus keptosis* as a stem lineage for (*Oncorhynchus gorbuscha*, *Oncorhynchus keta*).

The origin of Salmonidae was estimated to have occurred 59.1 MY ago (CI: 58.1–63.2 MY). To estimate the mitochondrial rate of molecular divergence across genera, we calibrated the genetic distances in the cytochromes matrix (corrected using a model of molecular evolution with 6 parameters (GTR+G+I)) with the inferred time since divergence. The average mitochondrial rate of molecular divergence across genera in Salmonidae for the two mitochondrial genes was estimated at 0.31%/MY (CI: 0.27–0.36%/MY). Notably, these rates vary among genera as well as genes, which resulted in the rejection of a molecular clock model.

The most recent split between genera would have occurred between *Brachymystax* and *Hucho* (19.9 MY; CI: 22.5-16.8 MY), which was slightly more recent than the split between *Oncorhynchus* and *Salvelinus* (25.1 MY; CI: 26.4-23.6 MY). Most intraspecific divergence occurred during the Pleistocene, although some earlier divergence times were suggested. The oldest intraspecific divergence time occurred in the Miocene in *Thymallus*, at 21.6 MY (*Thymallus grubii*), 10.6 MY (*Thymallus arcticus*) and 5.6 MY (*Thymallus thymallus*), while divergence between conspecific samples for *Hucho taimen* (4.1MY), the two geographically most distant lineages of *Coregonus clupeaformis* (2.7MY) and *Oncorhynchus clarkii* (2.1MY) occurred in the Pliocene. Recent interspecific divergence times reflect ambiguity associated with the species-level designations of some groups (e.g., Great Lakes ciscoes, *Coregonus lavaretus*, Bear Lake *Prosopium*, *Salvelinus alpinus*, *Oncorhynchus mykiss*). *Coregonus pollan* and *Salmo marmoratus* also show very recent divergence estimates, such that they were removed from the temporal calibration analysis due to their weak differentiation from closely related species.

## Discussion

Relative to previous studies, we substantially increased both the number of taxa and loci in an attempt to elucidate a more complete picture of the evolutionary relationships within Salmonidae. Thus, the most complete representation of Salmonidae presented here resolves some issues regarding the intergeneric relationships in the family and the three monotypic genera: *Parahucho* is a valid genus and is sister to (*Salvelinus*, *Oncorhynchus*); *Salvethymus* grouped within *Salvelinus* in all analyses and should be included within that genus; and *Stenodus* does not warrant its own genus and should be included within *Coregonus*. Second, we resolve many ambiguities and highlight some of the causes of the persistent difficulties associated with notoriously problematic relationships within the genera of Salmonidae, particularly within *Oncorhynchus*, *Coregonus* and *Salvelinus*. Third, our work supports that Thymallinae, rather than Coregoninae, is the sister group to the remainder of the family. Our results also support a much older history for some events of Salmonidae evolution than previously assumed, with an estimated family age of 59.1 MY (CI: 63.2-58.1 MY) and an average mitochondrial rate of divergence of ∼0.31%/MY. Nevertheless, several relationships remain unsolved, particularly within *Oncorhynchus*, *Salvelinus* and *Coregonus*.

Increasing the number of taxa sampled as well as the number of characters allowed us to increase confidence in our phylogenetic reconstruction and shed some light on existing questions in salmonid phylogeny. In addition to increasing taxa and characters sampled, using different types of inference increased confidence in some nodes that have been historically disputed. For instance, both parsimony and likelihood approaches suggested the same relationship among genera within Salmoninae using the MitoNuc-NT supermatrix, which was only possible due to the increased number of taxa sampled. Furthermore, functional recoding allowed for better detection and minimization of the sources of systematic errors that could have otherwise been entirely attributed to genomic conflicts. Indeed, analyses using both -NT and -RY data sets for MitoNuc and MitoNuc25 indicated that pyrimidine-purine re-coding resolved a large number of errors due to compositional bias and/or mutational saturation that were present mainly in the mitochondrial partition of the supermatrices. Maximum parsimony analyses are generally more susceptible to systematic errors than likelihood based criteria [74]–[78], and thus it is not surprising that the two types of inference converged on the same topology only after biases were minimized by functional recoding. Despite evidence for systematic errors and some conflict between mitochondrial and nuclear genomes (e.g. *Salmo* and *Salvelinus*; Figure 2), it is noteworthy that Bayesian analyses did not detect these sources of error in some cases. For example, in *Oncorhynchus*, nodes obtaining bootstrap values of 49% and 63% with ML had Bayesian posterior values of 100% (Figure 4B). This was also evident in the conflicting results for *Salvelinus*, suggesting that one must be cautious when interpreting posterior probabilities [79], [80].

### Intergeneric relationships and monotypic genera

Our results strongly suggest the following evolutionary relationship for Salmoninae:

((*Brachymystax*, *Hucho*), (*Salmo*, (*Parahucho*, (*Salvelinus*, *Oncorhynchus*))

This relationship differs slightly from that of Crespi and Fulton [5], where *Parahucho perryi* was often grouped with *Salmo*. In all of our supermatrix analyses, *Parahucho perryi* was sister to the (*Salvelinus*, *Oncorhynchus*) group, corroborating other findings [4], [10], [21], [22], [31], [81]. Strong support for the genus validity of *Parahucho* and its sister taxa designation with (*Salvelinus*, *Oncorhynchus*) was obtained both in the maximum likelihood (99% bootstrap value) and Bayesian (100% posterior probability) analyses on MitoNuc-NT despite the conflicting genomic signals detected in the MP analysis on that supermatrix.

In contrast with our finding that *Parahucho* is likely a true monotypic genus, our results for *Stenodus* and *Salvethymus* suggested that these genera may require taxonomic revision. For both genera, bootstrap and Bayesian posterior values were relatively low in all analyses, indicating that their position within their respective subfamilies was uncertain. However, in both cases, *Stenodus* and *Salvethymus* were found nested within *Coregonus* and *Salvelinus*, respectively, which indicates that they belong within these other genera despite a lack of precise evolutionary relationships. Thus, our results support previous findings that genus designation is not required for *Stenodus* [1], [26], [27] or *Salvethymus* [4], [25].

### Phylogenetic relationships within problematic genera

#### Coregonus

Within *Coregonus*, issues with species identification arise due to a complex evolutionary history [82], [83]. The main problem with identification arises from the diversity within two phenotypes, the whitefishes and the ciscoes. While these two phenotypic groups were considered monophyletic subgenera [82], molecular evidence suggests otherwise. For example, the Baïkal omul, *Coregonus migratorius*, has morphological features that may suggest that it is a cisco, but is more closely related to whitefish based on molecular similarity [84], [85]. Thus, based on morphological classification, the whitefish may be considered a paraphyletic group. Molecular evidence reveals that ciscoes are also a paraphyletic group [27], with the least cisco, *Coregonus sardinella*, being more closely related to the whitefish than other ciscoes. Our work supports this observation, which was reinforced by repeating our analyses excluding the *Coregonus tugun* sample, resulting in an increase in bootstrap support for the monophyly of the whitefishes with *C. sardinella*. The exclusion of *C. tugun* from our analyses also suggested a novel position for *Coregonus huntsmani* as the sister species to the rest of *Coregonus*. Previous studies have indicated that *C. huntsmani* represents a distinct evolutionary lineage [27], [86], however they did not find *C. huntsmani* to occupy that position in the genus. Several studies have previously observed the affinities of *Stenodus* with *Coregonus* [1], [26], [27], but our results clearly emphasize a definite need for a taxonomic revision for inclusion in the genus. Interestingly, *C. tugun* was solely responsible for a quarter of the unsupported nodes in the cytochromes analyses. The uncertain position of this small cisco, sometimes with whitefishes, the sardine cisco group or associated with nodes at the base of the genus, will therefore require more data to be resolved.

Many weakly supported nodes in our analyses are found in more recent splits in *Coregonus*. For example, relationships among the species in the *Coregonus artedi* complex (*Coregonus artedi*, *Coregonus hoyi*, *Coregonus kiyi*, *Coregonus nigripinnis* and *Coregonus zenethicus*) did not obtain robust support in any analysis, which may not be surprising given their very recent origin and evidence of patterns of reticulated evolution in these species [39], [87]–[89]. Similarly, weak support was obtained for the split between *Coregonus pollan* and *Coregonus autumnalis*, which confirms previous observations suggesting that these taxa may not be strongly differentiated and are possibly conspecific [90].

#### Salvelinus

Despite an increase in character and taxon sampling, relationships among *Salvelinus* species remain uncertain due to contradictory signals between mitochondrial and nuclear genes, as well as potential systematic errors in phylogeny reconstruction that were brought to light through RY-recoding (Figure 4). The recent divergence of many *Salvelinus* species, particularly those belonging to the most recently diverged *Salvelinus alpinus* clade, may explain issues with taxonomic and phylogenetic difficulties in this group. The *Salvelinus alpinus* group, consisting of *Salvelinus alpinus*, *Salvelinus malma*, *Salvelinus albus*, *Salvelinus elgyticus*, *Salvelinus boganidae* and *Salvelinus confluentus*, represents a large diversity of forms within and among species that are often found in sympatry [2], [91]. In addition, convergent evolution among these groups may occur due to the formation of similar ecological niches following glacial retreat. Contradictory signals between mitochondrial and nuclear genes indicate a major source of difficulty for phylogenetic inference in this genus, which is further complicated by hybridization between recently diverged species [4], [7], [33], [34], [69], [92]–[95]. The association between *Salvelinus confluentus* and the rest of the *Salvelinus alpinus* group based on mitochondrial genes was not supported by nuclear data, which corroborates numerous lines of evidence indicating introgression of the Arctic char mitochondrial genome in this species that may mask a sister taxon relationship with *Salvelinus leucomaenis* [4], [96]–[99].

#### Oncorhynchus

If we consider all results of the topology tests, only one of 15 possible configurations was non-significant for all eight analyses (relationship 11 in Table 1). The highest likelihood value for all analyses was consistent (relationship 1 in Table 1) and mirrors the relationship within *Oncorhynchus* shown in Figure 3 for which the highest support was obtained with RY-coding (Figure 4c and d). The ambiguous results obtained here reflect an historical difficulty with elucidating relationships in *Oncorhynchus*, which likely persist because of the rapid species radiation that occurred in this genus shortly following establishment (Figure 6). Interestingly, only one test (SH for MitoNuc25-NT) was significant for the sister taxa relationship between the Pacific trout and the Japanese salmon, a clade strongly supported in Crespi and Fulton's [5] Bayesian supermatrix analysis (2004). Despite the more exhaustive taxa and gene sampling presented here, an unequivocal portrait of *Oncorhynchus* seems more difficult to obtain than previously thought.

### Rooting Salmonidae

This study calls into question the general assertion that Coregoninae is the sister group to the remainder of Salmonidae. Instead, our analyses support the findings of a recent molecular study [13] that suggests that the root of Salmonidae may be at the base of Thymallinae. This result is also supported by allozyme data [100] and the relatively large number (2n = 98–102) of chromosomes in Thymallinae compared to other species in the family (2n = 52–92), which may be considered a retention of an ancestral trait [10], [101]. The hypothesis that Thymallinae was the first lineage to diverge from the ancestral node in Salmonidae has often been rejected due to an absence of both the orbitosphenoid bone (also absent in Esociformes) and the basibranchial plate, as well as morphological similarities with Salmoninae. Coregoninae is commonly accepted as the first group in the family to have diverged from the ancestral node due to a lack of teeth, although this character is otherwise observed in *Stenodus leucichthys*. Furthermore, vestigial teeth are present in a number of coregonine species [1], [3]. Our finding that Salmonidae may root at Thymallinae can also be partly attributed to the selection of Esociformes as the most appropriate outgroup [19], [20], [70], although Koop et al. [13] obtained the same result in their study using another outgroup.

### Divergence times

Our estimate of the age of Salmonidae of 59.1 MY (CI: 58.1–63.2 MY) is consistent with broad-scale analyses of phylogenetic relationships among fishes [106]–[107] and jawed vertebrates [108]. Divergence times throughout our phylogeny are generally older than those estimated by some previous studies [27], [31], [102]–[105]. It is important to interpret these estimates with caution. While the time estimates were not substantially affected by the evolutionary relationship of subfamilies inferred by the rooting analysis, our temporal analysis is clearly dependent upon the topological position and minimum fixed age of †*Eosalmo driftwoodensis* assumed to be at 50 MY ago. Although that calibration point was consistent with other fossils in the cross-validation analysis, it is especially difficult to determine the accuracy of the divergence timings deep in the tree, as independent estimates are scarce. In a recent study, Wilson and Turner [105] estimated that *Salmo* split from *Oncorhynchus* and *Salvelinus* between 13.9 and 24.0 MY ago based on a constrained divergence time of 15–20 MY ago between *Salmo* and *Oncorhynchus* for which there is no convincing fossil evidence. Our estimate, based solely on the fossil record, suggests an older split at approximately 26–29 MY ago. Ideally, an accurate reconstruction of divergence times would cross-validate fossil ages with specimens belonging to a number of different genera, as the fossil evidence used here was based only upon †*Eosalmo driftwoodensis* and four fossils associated with *Oncorhynchus*. These comparisons may bias rate estimates for other lineages due to strong heterogeneity in the rates of molecular evolution seen across the family. For more recent divergences (<1–2 MY), it is equally possible that differences between contemporary and historical evolutionary rates may result in age overestimation for young lineages [109], [110]. Our estimates of the average mitochondrial rate of molecular divergence (0.31%/MY; CI: 0.27–0.36%/MY) are considerably slower than the 1%/MY suggested by Smith [111]. However, this rate is likely dependent upon the timing of divergence, such that faster rates may be more applicable to comparisons involving recent speciation events. Given that our estimates are based on much deeper divergence for Salmonidae (∼59 MY), a relatively slow rate of change is not entirely unexpected because rates of molecular change tend to decay exponentially over time [110]. Indeed, our estimate is close to the lower bound of the range (0.34%/MY–1.7%/MY) estimated for other groups of fishes with divergence times between 5 and 15 MY [110]. In addition, divergence rates tended to vary strongly among genes and lineages, suggesting that estimates of divergence rates across lineages and genes obtained using a single molecular clock should be interpreted with caution. For example, mean divergence rates inferred within *Oncorhynchus* varied from 0.42%/MY (CI: 0.39–0.45%/MY) for CO1 to 0.63%/MY (CI: 0.58–0.67%/MY) for Cytb. These estimates are nevertheless compatible with previous estimates in *Oncorhynchus*; McKay et al. [112] estimated a divergence rate of 0.83%/MY based on ND3, a gene with a relatively fast divergence rate among mitochondrial genes (Table S2), and Wilson and Turner [105] estimated divergence rate of 0.71%/MY based on ND4, a gene with a relative rate of molecular divergence similar to Cytb (Table S2). Furthermore, estimates of divergence rates vary across taxa. For instance, the divergence rates for *Oncorhynchus* stated above were double those for *Coregonus*, which ranged between 0.20%/MY (CI: 0.17–0.23%/MY) and 0.33%/MY (CI: 0.27–0.38%/MY).

## Conclusions

This study improved the portrait of Salmonidae by including twice as many species as previous morphological studies and three times more taxa than previous molecular studies, proposing a new evolutionary relationship of the family, providing more robust inferences for the relationships among Salmoninae genera, offering some insight into conflicts regarding different hypotheses for salmonid evolution and suggesting that the family may be much older than previously thought. However, many evolutionary relationships could not be resolved because radiation and hybridization may have eroded historical phylogenetic signals, particularly in *Oncorhynchus* and *Salvelinus*. From an evolutionary perspective, repeated hybridization may represent an important driver of diversification in the family. In complementary analyses, we found clues for hybridization in most genera, with the exception of the least diverse groups *Brachymystax*, *Hucho*, *Prosopium* and *Thymallus*. It is important to note that the gene tree depicted in this work may not be an accurate representation of the ‘true’ evolutionary relationships among species [113], [114], which may never be fully resolved. Despite these uncertainties, this work represents the most comprehensive analysis and provides the most complete picture of the evolution of the Salmonidae family to date.

## Materials and Methods

### Mitochondrial genotyping

Samples that were not obtained as genomic DNA were extracted from fin or muscle tissue using a DNeasy Tissue Kit (Qiagen Inc.). These samples were collected from the field and produced under the compliance and authorization of the Comité de protection des animaux de l'Université Laval, Québec, Canada, who approved sample collection for this study. The entire cytochrome b gene (Cytb; 1141 bp) and a segment of the 5′ end of the cytochrome c oxidase I gene (CO1; 1262 bp) were amplified using the following primer sets: CO1: 5′-TCA ACC AAC CAC AAA GAC ATT GGC AC [115] and 5′-AGT GTT TCA CAG TGT GTA GGC; Cytb: 5′-CAT AAT TCC TGC CCG GAC TCT AAC C and 5′-TTT AAC CTC CGA TCT CCG GAT TAC A. Reactions occurred in a 50 µL volume with 5 µL of genomic DNA (10–50 ng), 5 µL of 10× reaction buffer (500 mM KCl, 100 mM Tris-HCl (pH 9.0), 1.5 mM MgCl_2_, 1% Triton X-100), 4 µL of 2.5 mM dNTP, 20 pmol of each primer and 1 U of Taq polymerase. PCR conditions consisted of an initial denaturation at 95°C for 300 s, followed by 45 cycles of 95°C for 60 s, 51°C for 60 s and 72°C for 90 s. PCR products were run on 1.2% low-melting point agarose and fragments were excised from the gel prior to being purified using the QIAquick Gel Extraction Kit (QIAGEN). Fluorescent bidirectional sequencing was carried out by the Centre Hospitalier de l'Université Laval. Sequences were verified using PHRED [116] and edited with SeqLab and SeqMerge (Wisconsin Package v. 10.3; Accelrys (GCG)).

### Phylogenetic analysis of cytochrome genes

Nucleotide content, χ^2^ homogeneity tests and p-distances for each gene and codon position were calculated using PAUP* v.4b10 [117]. We inferred maximum parsimony (MP) trees for cytochromes using heuristic searches in PAUP* (TBR branch swapping, 1000 random stepwise taxon additions), from which a strict consensus tree was obtained. The robustness of the tree was evaluated by 1000 bootstrap pseudoreplicates using heuristic searches (50 random taxon additions). Optimal models of evolution were selected from 56 models of increasing complexity using ModelTest v. 3.7 [118], [119]. These models were subsequently used to infer phylogenetic relationships using maximum likelihood (ML) and Bayesian analysis (BAY). ML trees were constructed using the Pthreads version of RAxML v. 7.0.0 under the GTR+GAMMA+I model [120], [121] and robustness was assessed by bootstrapping 1000 times with the CAT approximation. Bayesian analysis of phylogenetic relationships was carried out using MrBayes v. 3.1.2 [122], [123] by partitioning sequences for each codon position and running the algorithm using a mixed model. Two analyses were run for 4×10^6^ generations with a random starting tree, and four Markov chains under default heating values, sampling every 100 generations. Stationarity of the MCMC analyses was determined by plotting −lnL against generation time and the “burn-in” trees sampled prior to stationarity were discarded. The consensus tree and posterior probabilities were determined from 60,000 sampled trees.

### Supermatrix construction

A supermatrix containing a maximum number of clusters of mitochondrial and nuclear sequences for Salmonidae was constructed using information in the PhyLoTA database [124]. A complete list of coding and non-coding sequences allowed for preliminary examination of candidate genes for the supermatrix. Sequences obtained from microsatellites, D-loop regions, transposons, mRNA and MHC were excluded from the supermatrix. In total, 52 genes, including 22 mitochondrial tRNAs, were identified by using an ‘all-against-all’ BLAST in GenBank (Release 160). A FASTA file was generated and edited for each of the 52 genes using Geneious 3.0.5 [125]. For each gene, a total of 45 alignments were carried out in ClustalW [126] using a range of parameter values (Gap Open: 3–15; Gap Extension: 3–7). The 45 alignments of a given gene were then compared in SOAP [127] to retain only stable nucleotide positions in the final alignments. Insertions/deletions of more than 2 bp were also excluded and the 22 tRNA sequences were concatenated into a single data partition. A total of 31 acceptable DNA sequence partitions were obtained, consisting of 17 nuclear genes, 13 mitochondrial genes and the concatenated tRNA sequences. These partitions were combined with the two genes of the cytochromes data set into a large supermatrix, MitoNuc-NT, comprising 33 gene partitions in total.

### Phylogenetic analysis of Supermatrix

Nucleotide content and χ^2^ homogeneity tests were performed in PAUP* for each gene in MitoNuc-NT. Maximum parsimony trees were constructed using similar parameter values and bootstrapping methods as were used for MP analysis of cytochromes alone. Clade support was determined using Templeton tests [128] and node support was assessed using Bremer support indices [129] according to whether genes were of nuclear or mitochondrial origin. Maximum likelihood (ML) trees for the supermatrix were inferred using RAxML under the GTRMIXI model. A second ML tree, assuming heterogeneous evolutionary processes underlie each gene, was inferred using PHYMLrates [130] to estimate evolutionary rates of each gene following an approximation generated in DistR [131], [132]. BAY trees were inferred using MrBayes by partitioning the matrix into genes that were assigned specific models of molecular evolution in ModelTest v. 3.7 (mixed model comprised of 33 individual models). These partition schemes were chosen as the best compromise between under- and over-parameterization of the models, while limiting the analyses to practical computation time considering the amount of data. Two analyses were run for 10×10^6^ generations with a random starting tree, and four Markov chains sampled every 1000 generations. The consensus tree and posterior probabilities were determined from 10,000 trees sampled after convergence to stationarity.

In order to minimize systematic errors in the phylogenetic inference of Salmonidae, the above analyses, with the exception of those using PHYMLrates, were also conducted on the supermatrix recoded in purine/pyrimidine (RY), hereafter referred to as MitoNuc-RY. For the BAY analyses, models of molecular evolution of MitoNuc-RY were determined using the first four models in ModelTest v.3.7 (testing for G, I and G+I) and a substitution model (NST = 1) for each group.

To evaluate the contribution of added taxa on the phylogenetic resolution, we created a smaller data set of 25 taxa, MitoNuc25-NT, forming an array of species comparable in scope to the 21 species found in Crespi and Fulton's [5] total evidence analysis. This data set was reduced to 31 gene partitions since two genes (RAG and Epend) had to be discarded from the analyses because they were too sparsely distributed or uninformative. The MitoNuc25-NT data set was analyzed using methods similar to those executed on MitoNuc-NT and was also re-coded in purine/pyrimidine (MitoNuc25-RY) to be analyzed as described above for MitoNuc-RY. Thus, we reconstructed the salmonid phylogeny on a total of four supermatrices using MP, ML and BAY methods.

### Topology tests

The two following groups were further evaluated by comparing all possible arrangements using topology tests: 1) the relationship among genera within Salmoninae; and 2) the evolutionary relationship for *Oncorhynchus* species. We conducted both AU [133] and SH [134] tests using CONSEL [135]. For these tests, topologically stable Salmoninae genera or *Oncorhynchus* species were placed into 5 groups and the 15 constrained trees representing all possible topologies for these groups were used to evaluate support for different evolutionary relationships.

### Rooting Salmonidae

To determine the root of the Salmonidae phylogenetic tree, different species were alternatively used as outgroups using a Bayesian approach [136], [137]. Outgroups were delimited based on recent molecular hypotheses [19], [20], [70] and consisted of four taxa: superfamily Alepocephaloidea (represented by *Alepocephalus tenebrosus* Gilbert 1892), superfamily Argentinoidea (represented by *Nansenia ardesiaca* Jordan and Thompson 1914); sub-order Osmeroidei (represented by *Galaxias maculatus* (Jenys 1842)); and order Esociformes (represented by *Esox lucius* Linnaeus 1758 and *Dallia pectoralis* Bean 1880). Complete mitochondrial genomes for representative outgroup species were obtained from GenBank and gene sequences were aligned simultaneously with the salmonid mitochondrial sequences using the same procedure used to construct the MitoNuc-NT supermatrix. Alignments were concatenated to the nuclear genes represented in MitoNuc-NT, resulting in four supermatrices each containing one of the outgroups. Those four supermatrices were also re-coded in purine/pyrimidine and trees were inferred using the same Bayesian procedures as for the supermatrices. The posterior probability distributions of the root based on the 10,000 post burn-in trees were then mapped on the unrooted Bayesian consensus phylogram. This procedure was repeated for each of the four outgroups.

### Temporal calibration of Salmonidae

We used a relaxed molecular clock to account for variable rates of evolution among lineages. Temporal reconstruction of the evolution of Salmonidae was carried out for the ML tree constructed on MitoNuc-NT using Penalized Likelihood (PL) with r8s [138], [139].

Following inference of absolute divergence times from relative substitution rates, we calibrated our phylogenetic tree using fossil evidence. Stearley and Smith's [3] matrix of 119 morphological characters that included four fossil species (†*Eosalmo driftwoodensis*, †*Salvelinus* (*Paleolox*) *larsoni*, †*Oncorhynchus* (*Rhabdofario*) *lacustris* and †*Oncorhynchus* (*Smilodonichthys*) *rastrosus*) was concatenated with MitoNuc-NT and analyzed using the same MP parameters for the analysis of MitoNuc-NT. The different positions of the fossils in the resulting MP tree suggested a total of six potential calibration nodes. In addition, the dated specimen of †*Oncorhynchus keptosis* [140] was placed as a minimum time constraint for (*Oncorhynchus nerka*, (*Oncorhynchus keta*, *Oncorhynchus gorbuscha*)) according to the authors description of the fossil. For all fossils, the best corroborated and/or the oldest dates were used for calibration [3], [18], [71]–[73], [110], [140]–[145].

To minimize potential issues with erroneous positioning of fossils within the tree topology [146], [147] or difficulty with accurately dating the appearance or geological position of fossil taxa, methods outlined by Near and Sanderson [148] and Near et al. [149] were used to validate the different calibration points. Three calibration points were retained for final analysis: a fixed age of 50 MY for †*Eosalmo driftwoodensis*, assigned as a stem lineage for Salmoninae; a minimum age of 11MY for †*Oncorhynchus* (*Smilodonichthys*) *rastrosus*, positioned as a stem lineage for (*Oncorhynchus masou*, (*Oncorhynchus nerka*, (*Oncorhynchus keta, Oncorhynchus gorbuscha*))); and a minimum age of 8MY for †*Oncorhynchus keptosis*, assigned as a stem lineage for (*O. nerka*, (*O. keta, O. gorbuscha*)). We also assigned minimum ages to two other fossils, despite age estimates that were less robust than the species mentioned above: a minimum age of 11MY for †*Salvelinus* (*Paleolox*) *larsoni*, positioned as a stem lineage for *Salvelinus*; and a minimal age of 3.2 MY assigned to †*Oncorhynchus* (*Rhabdofario*) *lacustris*, imposing a crown group age for the Pacific trout clade.

Cross-validation using 21 smoothing parameter values (0.1–10,000) was used on the four fossils with minimum age dates (†*Eosalmo driftwoodensis* was assigned a fixed date due to strong corroboration and support from previous studies [3], [18]) in order to find optimal λ values. A second cross-validation based on the fossils served to validate our first estimate using the *Fossil cross-validation* function in r8s [139], [149]. The λ parameter was then used in PL analysis to infer the chronogram of the family, which was re-run 10 times using the truncated Newton algorithm to avoid non-optimal solutions. To assess the possible impact of missing data in the MitoNuc-NT data set on the chronogram (63 species, 23% completeness), we conducted the preceding analyses on the MitoNuc25-NT ML tree as well (25 taxa, 52% completeness).

After evaluation of the reliability of date estimates, 100 bootstraps on the complete MitoNuc-NT data set was generated using SeqBoot in PHYLIP 3.6 [150]. Branch lengths were optimized using ML in PAUP* for each matrix using the evolutionary model determined by ModelTest. The PROFILE command in r8s allowed for the estimation of 95% confidence intervals of the age of the nodes.

## Supporting Information

Table S1 **Salmonidae specimens used in this study with collection site locations.**
(DOCX)

Table S2 **Genes, number of taxa and characteristics of sequences included in the MitoNuc supermatrix.**
(DOCX)
