# Supplementary material for: Framing the Salmonidae Family Phylogenetic Portrait: A More Complete Picture from Increased Taxon Sampling
Source: PLoS One. 2012 Oct 5;7(10):e46662. doi: 10.1371/journal.pone.0046662 (PMC3465342; doi:10.1371/journal.pone.0046662)
Supplement: Table S1 — Salmonidae specimens used in this study with collection site locations. (DOCX) [file pone.0046662.s001.docx]

Supplementary Table 1. Salmonidae specimens used in this study with collection site locations.

| **n^0^** | **Genus** | **Species** | **Vernacular name** | **Collection Site** | **Latitude** | **Longitude** |
| --- | --- | --- | --- | --- | --- | --- |
| **1** | *Brachymystax* | *lenok* (Pallas, 1773) | Lenok | Anui River, Russia (Amur basin) | 49°17' N | 137°55' E |
| **2** | *Brachymystax* | *lenok* (Pallas, 1773) | Lenok | Leprindokan Lake, Russia (Lena basin) | 56°33' N | 117°29' E |
| **3** | *Brachymystax* | *savinovi* Mitrofanov, 1959 | Lenok (blunt-snout form) | Amalyk Lake, Russia (Lena basin) | 57°35' N | 117°17' E |
| **4** | *Brachymystax* | *savinovi* Mitrofanov, 1959 | Lenok (blunt-snout form) | Anui River, Russia (Amur basin) | 49°17' N | 137°55' E |
| **5** | *Coregonus* | *albula* (Linnaeus, 1758) | Vendace | Lake Stechlin, Germany | 53°10' N | 13°02' E |
| **6** | *Coregonus* | *albula* (Linnaeus, 1758) | Vendace | Onega Lake, Russia | 61°30' N | 35°45' E |
| **7** | *Coregonus* | *artedi* Lesueur, 1818 | Cisco | Peerless Lake, Canada (AB) | 56°40' N | 114°30' O |
| **8** | *Coregonus* | *artedi* Lesueur, 1818 | Cisco | Lake Opasatica, Canada (QC) | 48°05' N | 79°15' O |
| **9** | *Coregonus* | *autumnalis* (Pallas, 1776) | Arctic cisco | Peel River, Canada (NWT) | 67°40' N | 134°38' O |
| **10** | *Coregonus* | *autumnalis* (Pallas, 1776) | Arctic cisco | Pechora River, Russia (Barents sea basin) | 67°39' N | 53°00' E |
| **11** | *Coregonus* | *clupeaformis* (Mitchill, 1818) | Lake whitefish | Cliff Lake, USA (ME) | 46°24' N | 69°15' O |
| **12** | *Coregonus* | *clupeaformis* (Mitchill, 1818) | Lake whitefish | Yukon River, USA (AK) | 65°20' N | 151°05' O |
| **13** | *Coregonus* | *hoyi* (Milner, 1874) | Bloater | Lake Superior, Canada (ON) | 48°10' N | 88°35' O |
| **14** | *Coregonus* | *hoyi* (Milner, 1874) | Bloater | Lake Huron, Canada (ON) | 44°41' N | 80°37' O |
| **15** | *Coregonus* | *huntsmani* Scott, 1987 | Atlantic whitefish | Milipsigate Lake, Canada (NS) | 44°20' N | 64°36' O |
| **16** | *Coregonus* | *kiyi* (Koelz, 1921) | Kiyi | Lake Superior, USA (WI) | 47°05' N | 90°30' O |
| **17** | *Coregonus* | *laurettae* Bean, 1881 | Bering cisco | Yukon River, USA (AK) | 61°52' N | 162°4' O |
| **18** | *Coregonus* | *laurettae* Bean, 1881 | Bering cisco | Yukon River, USA (AK) | 65°20' N | 151°05' O |
| **19** | *Coregonus* | *lavaretus* (Linnaeus, 1758) | Common whitefish | Lake Brienz, Switzerland | 46°44' N | 07°57' E |
| **20** | *Coregonus* | *migratorius* (Georgi, 1775) | Baikal omul | Lake Baïkal, Russia | 53°50' N | 109°05' E |
| **21** | *Coregonus* | *muksun* (Pallas, 1814) | Muksun | Ob' River, Russia | 66°31' N | 66°37' E |
| **22** | *Coregonus* | *nasus* (Pallas, 1776) | Broad whitefish | Sos'va River, Russia | 63°55' N | 65°01' E |
| **23** | *Coregonus* | *nasus* (Pallas, 1776) | Broad whitefish | Yukon River, USA (AK) | 65°20' N | 151°05' O |
| **24** | *Coregonus* | *nigripinnis* (Milner, 1874) | Blackfin cisco | Lake Nipigon, Canada (ON) | 49°45' N | 88°30' O |
| **25** | *Coregonus* | *peled* (Gmelin, 1789) | Northern whitefish | Ob' River, Russia | 66°31' N | 66°37' E |
| **26** | *Coregonus* | *pidschian* (Gmelin, 1789) | Humpback whitefish | Lyapin River, Russia | 63°48' N | 61°28' E |
| **27** | *Coregonus* | *pollan* Thompson, 1835 | Irish pollan | Lough Neagh, Ireland | 54°37' N | 6°23' O |
| **28** | *Coregonus* | *sardinella* Valenciennes, 1848 | Least cisco | Yukon River, USA (AK) | 65°20' N | 151°05' O |
| **29** | *Coregonus* | *sardinella* Valenciennes, 1848 | Least cisco | Shingle Point, Canada (NWT) | 68°59' N | 137° 23' O |
| **30** | *Coregonus* | *tugun* (Pallas, 1814) | Tugun | Sos'va River, Russia | 63°55' N | 65°01' E |
| **31** | *Coregonus* | *ussuriensis* Berg, 1906 | Amur whitefish | Heilongjiang province, China | - | - |
| **32** | *Coregonus* | *zenithicus* (Jordan & Evermann, 1909) | Shortjaw cisco | Lake Superior, USA (WI) | 47°05' N | 90°30' O |
| **33** | *Hucho* | *hucho* (Linnaeus, 1758) | Huchen | Drau River, Austria | 46°32' N | 14°21' E |
| **34** | *Hucho* | *taimen* (Pallas, 1773) | Taimen | Khor River, Russia (Amur drainage) | 47°45' N | 137°18' E |
| **35** | *Hucho* | *taimen* (Pallas, 1773) | Taimen | Bol'shaya River, Russia (Baikal-Enisei drainage) | 54°28' N | 109°29' E |
| **36** | *Oncorhynchus* | *apache* (Miller, 1972) | Apache trout | Soldier Creek, USA (AZ) | 34°05' N | 109°40' O |
| **37** | *Oncorhynchus* | *chrysogaster* (Needham & Gard, 1964) | Mexican golden trout | Durango, Mexico | - | - |
| **38** | *Oncorhynchus* | *clarkii* (Richardson, 1836) | Cutthroat trout | Red Deer Lake, Canada (AB) | 52°43' N | 113°03' O |
| **39** | *Oncorhynchus* | *clarkii* (Richardson, 1836) | Cutthroat trout | Birch Creek, USA (UT) | 39°53' N | 113°57' O |
| **40** | *Oncorhynchus* | *gilae* (Miller, 1950) | Gila trout | Spruce Creek, USA (NM) | 33°17' N | 108°42' O |
| **41** | *Oncorhynchus* | *gorbuscha* (Walbaum, 1792) | Pink salmon | Vedder River, Canada (BC) | 49°05' N | 121°58' O |
| **42** | *Oncorhynchus* | *gorbuscha* (Walbaum, 1792) | Pink salmon | Pillar Creek, USA (AK) | 57°48' N | 152°25' O |
| **43** | *Oncorhynchus* | *keta* (Walbaum, 1792) | Chum salmon | Nitinat River, Canada (BC) | 48°50' N | 124°39' O |
| **44** | *Oncorhynchus* | *keta* (Walbaum, 1792) | Chum salmon | Horonai Stream, Japan | 42°41' N | 141°30' E |
| **45** | *Oncorhynchus* | *kisutch* (Walbaum, 1792) | Coho salmon | Atnarko River, Canada (BC) | 52°23' N | 125°49' O |
| **46** | *Oncorhynchus* | *kisutch* (Walbaum, 1792) | Coho salmon | Big Qualicum River, Canada (BC) | 49°23' N | 124°36' O |
| **47** | *Oncorhynchus* | *masou* (Brevoort, 1856) | Cherry salmon | Japan | - | - |
| **48** | *Oncorhynchus* | *masou* (Brevoort, 1856) | Cherry salmon | Shumarinai Lake, Japon | 44°21' N | 142°13' E |
| **49** | *Oncorhynchus* | *mykiss* (Walbaum, 1792) | Rainbow trout | Isabella Lake, Canada (AB) | 51°47' N | 116°25' O |
| **50** | *Oncorhynchus* | *mykiss* (Walbaum, 1792) | Rainbow trout (Steelhead form) | Ninilchik River, USA (AK) | 60°01' N | 151°36' O |
| **51** | *Oncorhynchus* | *mykiss* (Walbaum, 1792) | Rainbow trout | Arroyo Nogales, Mexico (Durango) | 25°31' N | 105°00' O |
| **52** | *Oncorhynchus* | *nerka* (Walbaum, 1792) | Sockeye salmon | Skeena River, Canada (BC) | 54°07' N | 130°05' O |
| **53** | *Oncorhynchus* | *nerka* (Walbaum, 1792) | Sockeye salmon | USA (AK) | - | - |
| **54** | *Oncorhynchus* | *rhodurus* Jordan & McGregor, 1925 | Biwamasu (in Japanese) | Biwa Lake, Japan | 35°15' N | 136°05' E |
| **55** | *Oncorhynchus* | *tshawytscha* (Walbaum, 1792) | Chinook salmon | Skeena River, Canada (BC) | 54°07' N | 130°05' O |
| **56** | *Oncorhynchus* | *tshawytscha* (Walbaum, 1792) | Chinook salmon | Grover's Creek Hatchery, USA (WA) | 47°44' N | 122°38' O |
| **57** | *Parahucho* | *perryi* (Brevoort, 1856) | Japanese huchen | Sapporo city hatchery, Japan | 43°03' N | 141°20' E |
| **58** | *Prosopium* | *abyssicola* (Snyder, 1919) | Bear Lake whitefish | Bear Lake, USA (UT) | 41°57' N | 111°20' O |
| **59** | *Prosopium* | *coulterii* (Eigenmann & Eigenmann, 1892) | Pygmy whitefish | Lake Superior, Canada (ON) | 48°10' N | 88°35' O |
| **60** | *Prosopium* | *coulterii* (Eigenmann & Eigenmann, 1892) | Pygmy whitefish | Chapman Lake, Canada (BC) | 54°56' N | 126°40' O |
| **61** | *Prosopium* | *cylindraceum* (Pennant, 1784) | Round whitefish | Musquacook Lake, USA (ME) | 46°40' N | 69°11' O |
| **62** | *Prosopium* | *cylindraceum* (Pennant, 1784) | Round whitefish | Kanuti River, USA (AK) | 66°32' N | 151°16' O |
| **63** | *Prosopium* | *gemmifer* (Snyder, 1919) | Bonneville cisco | Bear Lake, USA (UT) | 41°57' N | 111°20' O |
| **64** | *Prosopium* | *gemmifer* (Snyder, 1919) | Bonneville cisco | Bear Lake, USA (UT) | 41°57' N | 111°20' O |
| **65** | *Prosopium* | *spilonotus* (Snyder, 1919) | Bonneville whitefish | Bear Lake, USA (UT) | 41°57' N | 111°20' O |
| **66** | *Prosopium* | *spilonotus* (Snyder, 1919) | Bonneville whitefish | Bear Lake, USA (UT) | 41°57' N | 111°20' O |
| **67** | *Prosopium* | *williamsoni* (Girard, 1856) | Mountain whitefish | Bear Lake, USA (UT) | 41°57' N | 111°20' O |
| **68** | *Prosopium* | *williamsoni* (Girard, 1856) | Mountain whitefish | Queens river, USA (ID) | 43°49' N | 115°12' O |
| **69** | *Salmo* | *marmoratus* Cuvier, 1829 | Soska postry (in Slovenian) | Zala River, Slovenia (Adriatic Sea basin) | 45°55' N | 14°05' E |
| **70** | *Salmo* | *obtusirostris* (Heckel, 1851) | Adriatic trout (Krkensis form) | Krkić River, Croatia (Adriatic Sea Basin) | 44°05' N | 16°12' E |
| **71** | *Salmo* | *obtusirostris* (Heckel, 1851) | Adriatic trout (Oxrhynchus form) | Buna River, Bosnia-Herzegovena | 43°15' N | 17°50' E |
| **72** | *Salmo* | *ohridanus* Steindachner, 1892 | Belushka (in Albanian) | Lake Ohrid, Republic of Madedonia | 41°05' N | 20°45' E |
| **73** | *Salmo* | *ohridanus* Steindachner, 1892 | Belushka (in Albanian) | Lake Ohrid, Republic of Madedonia | 41°05' N | 20°45' E |
| **74** | *Salmo* | *platycephalus* Behnke, 1968 | Flathead trout | Soğuksu stream, Turkey | 40°51' N | 30°53' E |
| **75** | *Salmo* | *salar* Linnaeus, 1758 | Atlantic salmon | Rivière Ste-Marguerite, Canada (QC) | 48°20' N | 70°00' O |
| **76** | *Salmo* | *salar* Linnaeus, 1758 | Atlantic salmon | Ims, Norway (Atlantic Ocean ) | 58°54' N | 5°57' E |
| **77** | *Salmo* | *trutta* Linnaeus, 1758 | Brown trout (Atlantic lineage) | Leksa River, Norway (Atlantic Ocean basin) | 63°20' N | 10°55' E |
| **78** | *Salmo* | *trutta* Linnaeus, 1758 | Brown trout (Danubian lineage) | Arpa River, Armenia (Caspian Sea basin) | 39°40' N | 45°35' E |
| **79** | *Salmo* | *trutta* Linnaeus, 1758 | Brown trout (Mediterranean lineage) | Voidomatis River, Greece (Adriatic Sea basin) | 39°55' N | 20°40' E |
| **80** | *Salmo* | *trutta* Linnaeus, 1758 | Brown trout (Adriatic lineage) | Göksu River, Turkey (Mediterranean Sea basin) | 36°40' N | 32°38' E |
| **81** | *Salvelinus* | *albus* Glubokovsky, 1977 | White char | Kamchatka River, Russia | 56°04' N | 159°52' E |
| **82** | *Salvelinus* | *alpinus* (Linnaeus, 1758) | Arctic char | Lake Paul, Canada (QC) | 48°59' N | 64°59' O |
| **83** | *Salvelinus* | *alpinus* (Linnaeus, 1758) | Arctic char | Fjellfrøssvatnet lake, Norway | 69°05' N | 19°22' E |
| **84** | *Salvelinus* | *alpinus* (Linnaeus, 1758) | Arctic char | Lama Lake, Russie (Taimyr Peninsula) | 69°30' N | 90°10' E |
| **85** | *Salvelinus* | *boganidae* Berg, 1926 | Boganida char | El'gygytgyn Lake, Russia | 67°30' N | 172°05' E |
| **86** | *Salvelinus* | *confluentus* (Suckley, 1859) | Bull trout | Upper Arrow Lake, Canada (BC) | 50°32' N | 117°57' O |
| **87** | *Salvelinus* | *confluentus* (Suckley, 1859) | Bull trout | Metiolus River, USA (OR) | 44°31' N | 121°38' O |
| **88** | *Salvelinus* | *elgyticus* Viktorovsky & Glubokovsky, 1981 | Small-mouth char | El'gygytgyn Lake, Russia | 67°30' N | 172°05' E |
| **89** | *Salvelinus* | *fontinalis* (Mitchill, 1814) | Brook trout | Rivière Nouvelle, Canada (QC) | 48°07' N | 66°17' O |
| **90** | *Salvelinus* | *fontinalis* (Mitchill, 1814) | Brook trout | Lake William, Canada (QC) | 46°47' N | 72°53' O |
| **91** | *Salvelinus* | *leucomaenis* (Pallas, 1814) | Whitespotted char | Japon | - | - |
| **92** | *Salvelinus* | *leucomaenis* (Pallas, 1814) | Whitespotted char | Belaya River, Russia | 47°20' N | 143°00' E |
| **93** | *Salvelinus* | *malma* (Walbaum, 1792) | Dolly Varden | Harrisson Lake, Canada (AB) | 51°17' N | 115°57' O |
| **94** | *Salvelinus* | *malma* (Walbaum, 1792) | Dolly Varden | Kamchatka river, Russia | 54°10' N | 158°01' E |
| **95** | *Salvelinus* | *namaycush* (Walbaum, 1792) | Lake trout | Lac Argile, Canada (QC) | 45°46' N | 75°35' O |
| **96** | *Salvelinus* | *namaycush* (Walbaum, 1792) | Lake trout | Twin Lake, USA (MT) | 45°24' N | 113°42' O |
| **97** | *Salvethymus* | *svetovidovi* Chereshnev & Skopets, 1990 | Long-finned char | El'gygytgyn Lake, Russia | 67°30' N | 172°05' E |
| **98** | *Stenodus* | *leucichthys* (Güldenstädt, 1772) | Inconnu | Yukon River, USA (AK) | 65°20' N | 151°05' W |
| **99** | *Thymallus* | *arcticus* (Pallas, 1776) | Arctic grayling | Davatchan River, Russia (Lena drainage) | 56°33' N | 117°29' E |
| **100** | *Thymallus* | *arcticus* (Pallas, 1776) | Arctic grayling | USA (MT) | - | - |
| **101** | *Thymallus* | *arcticus baicalensis* (Dybowski, 1874) | Baikal grayling | Lake Baïkal, Russia | 55°41' N | 109°53' E |
| **102** | *Thymallus* | *brevirostris* Kessler, 1879 | Mongolian grayling | Kokh Nuur, Mongolia | 47°31' N | 98°27' E |
| **103** | *Thymallus* | *brevirostris* Kessler, 1879 | Mongolian grayling | Kokh Nuur, Mongolia | 47°31' N | 98°27' E |
| **104** | *Thymallus* | *grubii* Dybowski, 1869 | Amur grayling | Sypchegurka river, Russia | 51°20' N | 113°26' E |
| **105** | *Thymallus* | *grubii* Dybowski, 1870 | Amur grayling | Anui River, Russia | 49°17' N | 137°55' E |
| **106** | *Thymallus* | *thymallus* (Linnaeus, 1758) | Grayling | Möll River, Austria | 46°50' N | 13°23' E |
| **107** | *Thymallus* | *thymallus* (Linnaeus, 1758) | Grayling | Rivière Dessoubre, France | 47°15' N | 06°44' E |
