# Supplementary material for: Framing the Salmonidae Family Phylogenetic Portrait: A More Complete Picture from Increased Taxon Sampling
Source: PLoS One. 2012 Oct 5;7(10):e46662. doi: 10.1371/journal.pone.0046662 (PMC3465342; doi:10.1371/journal.pone.0046662)
Supplement: Table S2 — Genes, number of taxa and characteristics of sequences included in the MitoNuc supermatrix. (DOCX) [file pone.0046662.s002.docx]

Supplementary Table 2. Genes, number of taxa and characteristics of sequences included in the MitoNuc supermatrix.

| **Gene** | **No. samples** | **Length** | **No. variable sites** | **No. informative sites** | **Average Nucleotide frequency** | | | | **Comp. bias^a^** | **Evolutionary model** | **Gamma** | **P-invariant** | **Relative evolutionary rate^b^** |
| --- | --- | --- | --- | --- | --- | --- | --- | --- | --- | --- | --- | --- | --- |
|  |  | **(bp)** | **bp (proportion)** | **bp (proportion)** |  |  |  |  |  |  |  |  |  |
| *Mitochondrial* |  |  |  |  | A | C | G | T |  |  |  |  |  |
|  |  |  |  |  |  |  |  |  |  |  |  |  |  |
| 12S | 16 | 940 | 102 (0.11) | 58 (0.06) | 0.30 | 0.27 | 0.23 | 0.20 |  | GTR+I+G | 0.585 | 0.723 | 0.346 |
| 16S | 22 | 1683 | 199 (0.12) | 110 (0.07) | 0.32 | 0.25 | 0.23 | 0.20 | ** | TVM+I+G | 0.666 | 0.750 | 0.435 |
| ATP6 | 30 | 684 | 248 (0.36) | 221 (0.32) | 0.26 | 0.32 | 0.13 | 0.29 | * | TIM+I+G | 1.638 | 0.607 | 1.674 |
| ATP8 | 11 | 168 | 25 (0.15) | 9 (0.05) | 0.29 | 0.33 | 0.13 | 0.25 |  | K81uf+G | 0.227 | - | 0.476 |
| CO1 | 107 | 1551 | 500 (0.32) | 457 (0.30) | 0.23 | 0.27 | 0.19 | 0.31 |  | GTR+I+G | 1.735 | 0.649 | 1.434 |
| CO2 | 11 | 686 | 148 (0.22) | 93 (0.14) | 0.27 | 0.28 | 0.17 | 0.28 |  | TVM+I+G | 0.785 | 0.614 | 1.024 |
| CO3 | 12 | 786 | 212 (0.27) | 129 (0.16) | 0.24 | 0.29 | 0.17 | 0.29 | ** | TIM+I+G | 1.083 | 0.618 | 1.392 |
| Cytb | 107 | 1141 | 433 (0.38) | 413 (0.36) | 0.24 | 0.30 | 0.16 | 0.29 | ** | TIM+I+G | 1.074 | 0.580 | 1.923 |
| ND1 | 24 | 964 | 356 (0.37) | 306 (0.32) | 0.25 | 0.30 | 0.16 | 0.29 | ** | GTR+I+G | 1.110 | 0.562 | 1.981 |
| ND2 | 11 | 1050 | 394 (0.38) | 240 (0.23) | 0.27 | 0.32 | 0.13 | 0.27 | * | GTR+I+G | 1.630 | 0.529 | 2.142 |
| ND3 | 19 | 351 | 143 (0.40) | 115 (0.33) | 0.23 | 0.32 | 0.16 | 0.29 |  | GTR+I+G | 0.871 | 0.446 | 2.144 |
| ND4 | 11 | 1381 | 467 (0.34) | 299 (0.22) | 0.27 | 0.30 | 0.15 | 0.28 | * | TIM+I+G | 2.686 | 0.607 | 1.996 |
| ND4L | 12 | 297 | 73 (0.25) | 44 (0.15) | 0.24 | 0.32 | 0.17 | 0.27 |  | TVM+I+G | 0.446 | 0.465 | 1.377 |
| ND5 | 14 | 1832 | 627 (0.34) | 418 (0.23) | 0.29 | 0.30 | 0.14 | 0.27 | ** | GTR+G | 0.216 | - | 1.812 |
| ND6 | 13 | 522 | 191 (0.37) | 124 (0.24) | 0.14 | 0.14 | 0.35 | 0.37 |  | HKY+I+G | 0.727 | 0.452 | 2.012 |
| mtDNA/tRNA^c^ | 34 | 1561 | 185 (0.12) | 84 (0.05) | 0.28 | 0.23 | 0.23 | 0.27 |  | TVM+I+G | 0.614 | 0.738 | N/A |
| **Sum** |  | **15597** | **4303 (0.23)** | **3120 (0.20)** | **-** | **-** | **-** | **-** | ****** | **-** | **-** | **-** | **-** |
| **Average** |  | **975** | **269** | **195** | **0.26** | **0.28** | **0.18** | **0.28** | **-** | **-** | **-** | **-** | **1.478** |
| *Nuclear* |  |  |  |  |  |  |  |  |  |  |  |  |  |
| 18S | 8 | 1823 | 73 (0.04) | 5 (0.003) | 0.24 | 0.25 | 0.29 | 0.23 |  | HKY | - | - | 0.144 |
| CT | 5 | 96 | 4 (0.04) | 2 (0.02) | 0.26 | 0.34 | 0.24 | 0.16 |  | HKY | - | - | 0.306 |
| Epend | 4 | 462 | 44 (0.10) | 5 (0.01) | 0.26 | 0.20 | 0.19 | 0.36 |  | HKY | - | - | 0.531 |
| GH1c | 23 | 811 | 300 (0.37) | 149 (0.18) | 0.30 | 0.23 | 0.18 | 0.29 |  | K81uf+G | 0.943 | - | 1.016 |
| GH1d | 24 | 1151 | 402 (0.35) | 177 (0.15) | 0.32 | 0.19 | 0.18 | 0.30 |  | TVM+G | 1.318 | - | 0.685 |
| GH2c | 24 | 640 | 154 (0.24) | 75 (0.12) | 0.33 | 0.22 | 0.16 | 0.29 |  | TVM+I | - | 0.242 | 0.659 |
| GH2d | 27 | 1199 | 407 (0.34) | 188 (0.16) | 0.32 | 0.19 | 0.17 | 0.32 |  | TVM+G | 1.495 | - | 0.653 |
| HMG1 | 4 | 547 | 19 (0.04) | 3 (0.006) | 0.32 | 0.23 | 0.19 | 0.27 |  | HKY | - | - | 0.217 |
| ITS1 | 31 | 462 | 183 (0.40) | 135 (0.29) | 0.15 | 0.34 | 0.32 | 0.20 |  | GTR+G | 1.151 | - | 1.118 |
| ITS2 | 24 | 333 | 107 (0.32) | 63 (0.19) | 0.13 | 0.34 | 0.32 | 0.21 | ** | TVM+G | 0.403 | - | 0.823 |
| LDH | 7 | 384 | 48 (0.13) | 15 (0.04) | 0.25 | 0.23 | 0.18 | 0.33 |  | HKY | - | - | 0.529 |
| MetA | 9 | 491 | 95 (0.19) | 22 (0.05) | 0.26 | 0.21 | 0.21 | 0.32 |  | K81uf+G | 1.419 | - | 0.553 |
| MetB | 8 | 935 | 201 (0.22) | 42 (0.05) | 0.27 | 0.21 | 0.21 | 0.31 |  | TVM+I | - | 0.427 | 0.812 |
| RAG | 4 | 1556 | 105 (0.07) | 20 (0.01) | 0.22 | 0.28 | 0.31 | 0.19 | ** | TIM | - | - | 0.192 |
| Tnfa | 4 | 529 | 34 (0.06) | 11 (0.02) | 0.28 | 0.20 | 0.22 | 0.30 |  | HKY+I | - | 0.756 | 0.436 |
| Transf | 7 | 922 | 78 (0.09) | 35 (0.04) | 0.24 | 0.21 | 0.24 | 0.31 |  | TrN+G | 0.449 | - | 0.825 |
| VIT | 11 | 1488 | 199 (0.13) | 77 (0.05) | 0.29 | 0.19 | 0.21 | 0.31 |  | TVM+G | 0.536 | - | 0.333 |
| **Sum** |  | **13829** | **2453 (0.18)** | **1024 (0.08)** | **-** | **-** | **-** | **-** | **-** | **-** | **-** | **-** | **-** |
| **Average** |  | **813** | **144** | **60** | **0.26** | **0.24** | **0.23** | **0.28** | **-** | **-** | **-** | **-** | **0.578** |
| *Combined* |  |  |  |  |  |  |  |  |  |  |  |  |  |
| **Sum** |  | **29426** | **6756 (0.23)** | **4144 (0.14)** | **-** | **-** | **-** | **-** | ****** | **GTR+I+G** | **0.754** | **0.545** | **-** |
| **Average** |  | **892** | **205** | **126** | **0.26** | **0.26** | **0.20** | **0.28** | **-** | **-** | **-** | **-** | **1.000** |

^a^ Compositional bias. *-probability <0.05; **-probability<0.01.

^b^ Determined after optimization of the ML tree topology by PhyMLrates.

^c^ Transfer RNA was concatenated to a single sequence.
